# Chemical and Metabolic Profiling of Si-Ni Decoction Analogous Formulae by High performance Liquid Chromatography-Mass Spectrometry

**DOI:** 10.1038/srep11638

**Published:** 2015-06-29

**Authors:** Qian Chen, Shun Xiao, Zhenhao Li, Ni Ai, Xiaohui Fan

**Affiliations:** 1 Pharmaceutical Informatics Institute, College of Pharmaceutical Sciences, Zhejiang University, Hangzhou 310058, China

## Abstract

Along with an indispensable role in healthcare system of China for centuries, Traditional Chinese Medicine (TCM) shows increasing usages as complementary therapy in western countries. To improve our understanding on their therapeutic effects, it’s critical to unveil chemical compositions of TCM formula, the predominant form of therapy in TCM. However, intrinsic chemical complexity makes it a challenging task to perform analysis on each individual TCM formula even with most current state-of-art analytic techniques available. In this work we approached this question by focusing on analogous formulae, a unique category of TCM formulae grouped together based on shared herbs and/or similar TCM syndromes. Systematic chemical profiling on five Si-Ni decoctions (SNs) for cardiovascular diseases was performed by multistage MS and high-resolution MS (HR-MS) experiments. A total of 83 compounds, including alkaloids, flavonoids, ginsenosides, bile acids and triterpenoids, were described. Analysis on SNs-treated rats detected 55 prototype compounds and 39 metabolites in the systemic circulation *in vivo*, which may contribute directly to their observed clinical efficacies. This approach offers great advantage to speed up identification of chemical compositions of formula and reveal the difference among these analogous formulae that may be related to diverse clinical effects.

For centuries Traditional Chinese Medicine (TCM) has been widely used to prevent and treat many common diseases in China and other Asian countries. Meanwhile, its popularity has continued growing as an complementary/alternative therapy in the West^1^. In accordance with ancient TCM compatibility theory originated from balance and harmony, different medicinal herbs (sometimes involving minerals and animal-related products) are organized together to establish a TCM formula for treating patient^2^. In a TCM formula the positive effects of multiple herbs are enhanced while negative side effects that may occur when used individually would be reduced or eliminated, which makes TCM formula a predominant form of TCM therapy to address traditional phenotypic syndromes of patients. Among TCM formulae, analogous formulae are a unique category on account of sharing some herbs and/or using for the same or similar syndrome with different symptoms and signs. Overlapped TCM composition in analogous formulae provides a great opportunity to investigate relationship between their chemical profiles and variations in clinical applications and unveil scientific foundation of TCM compatibility rules. This information would greatly improve our understanding on molecular mechanisms of TCM, therefore further promoting worldwide recognition about TCM therapy and facilitating its globalization. However, chemical analyses on analogous formulae remain limited, which partially relates to their intrinsic complexity.

In recent years, owing to rapid development in analytical technical advances and high availability of instrumentation, liquid chromatography-mass spectrometry (LC-MS) has become one of the most essential tools for the rapid analysis of TCM constituents. Compared with conventional phytochemistry techniques, HPLC-ESI-MS^n^ can achieve highly efficient separation and rich structural information in chemicals at the same time^3^. Then HR-MS data shows the accurate mass and relative ion abundance of the target peaks which makes it possible to calculate their potential elemental compositions according to the spectral isotope distribution on the basis of natural isotope abundance^4^. LC-MS is a powerful analytical method for identification of known compounds and elucidation of unknown compounds in TCM complex matrix^5^,^6^,^7^,^8^, such as TCM formulae. On account of constituent diversity, molecular complexity and limitations of analysis technics, only some relatively simple or classical TCM formulae have been clarified the characterization of their constituents. Wang *et al*. studied the chemical constituents in Da-Huang-Gan-Cao-Tang, a famous formula consisting of *Rhei Radix et Rhizoma* (Da Huang) and *Glycyrrhizae Radix et Rhizoma* (Gan Cao) in ratio of 4:1 *w/w* in Jin-Gui-Yao-Lue, using the combination of LC-ESI-Q-TOF-MS and LC-ESI-IT-MS^5^. 104 compounds were identified on the basis of their accurate molecular weight and multistage MS data. Because unclear chemical constituents in TCM formulae limits further pharmalogical research, systemic chemical profiling is an essential foundation to understand TCM formulae. Further, compounds absorbed into the systemic circulation and metabolites are important for the explanation of pharmacological efficacies of a TCM formula because oral administration is the traditional and classic dosing route for TCM formulae^9^. From pharmacokinetic point of view, only a fraction of components in the formulae absorbed into the blood would exert therapeutic effects *in vivo*. It is critical to identify these potential bioactive constituents responsible for bioactivities of TCM formulae^10^,^11^.

Si-Ni decoction, described in ancient Zhang Zhongjing’s herbal formulae (*Shanghan Lun*, which is a Chinese medical treatise compiled by Zhang Zhongjing to treat epidemic infectious diseases causing fevers), is a typical TCM formula which has been used for over 2000 years. It consists of *Aconiti lateralis radix* (Fuzi, *ranunculaceae*)*, Glycyrrhizae radix et rhizome praeparata cum melle* (Zhigancao, *Fabaceae*) and *Zingiberis rhizoma* (Ganjiang, *Zingibeaceae*). SNs includes Si-Ni decoction (SIN), Fuling Si-Ni decoction (FSIN), Ginseng Si-Ni decoction (RSIN), Tongmai Si-Ni decoction (TSIN) and Tongmai Si-Ni with *Suis fellis pulvis* decoction (ZSIN) with different content ratio or adding *Suis fellis pulvis* (Zhudanfen, *Sus scrofa domestica Brisson*) or *Ginseng radix et rhizoma* (Renshen, *Araliaceae*) and/or *Poria* (Fuling, *Polypores*). SNs have been used to prevent or treat cardiovascular disease for many years^12^,^13^,^14^,^15^, but their components are still poorly understood^16^. In this paper, an integrated approach by combining pre-classification strategy^17^ and diagnostic fragment-ion-based extension strategy^18^,^19^ was developed to characterize and compare the chemical components of SNs systematically *in vitro* and *in vivo* based on multistage MS and HR-MS (Fig. 1). The pre-classification strategy^17^ on multiple spectrums of TCM formula was previous proposed by our group on the basis that each class of nature products, such as flavonoids, alkaloids, and saponins, shares a characteristic carbon skeleton or the same structural units. It not only improved the accuracy but also sped up the identification of compounds from chemical databases and relevant literatures. Until now chemical differentiation study has not yet performed on these five formulae and very little is known about the chemical profiling of SNs, especially those *in vivo*. Results from this work will facilitate further research in pharmacological basis for clinical application of these five formulae.

## Results

### Validation of HPLC-MS chemical profiling method

To ensure the accurate assessment and comparison chemical profiling of SNs, nine constituents in SNs, which cover major structural groups, were selected as representatives with measuring the peak area and retention time. The repeatability and stability of the method were satisfactory with the peak area and retention time variations for nine constituents less than 9.21% and 0.35%, respectively. The method also provided good intra-day and inter-day precision. The RSDs of retention time and peak area of the nine constituents were in the range from 0.03% to 0.27% and 2.59% to 8.82%, respectively. According to the results, the proposed method is reliable and accurate for the qualitative analysis of SNs.

### Identification of chemicals in FSIN

**Figure 2:**
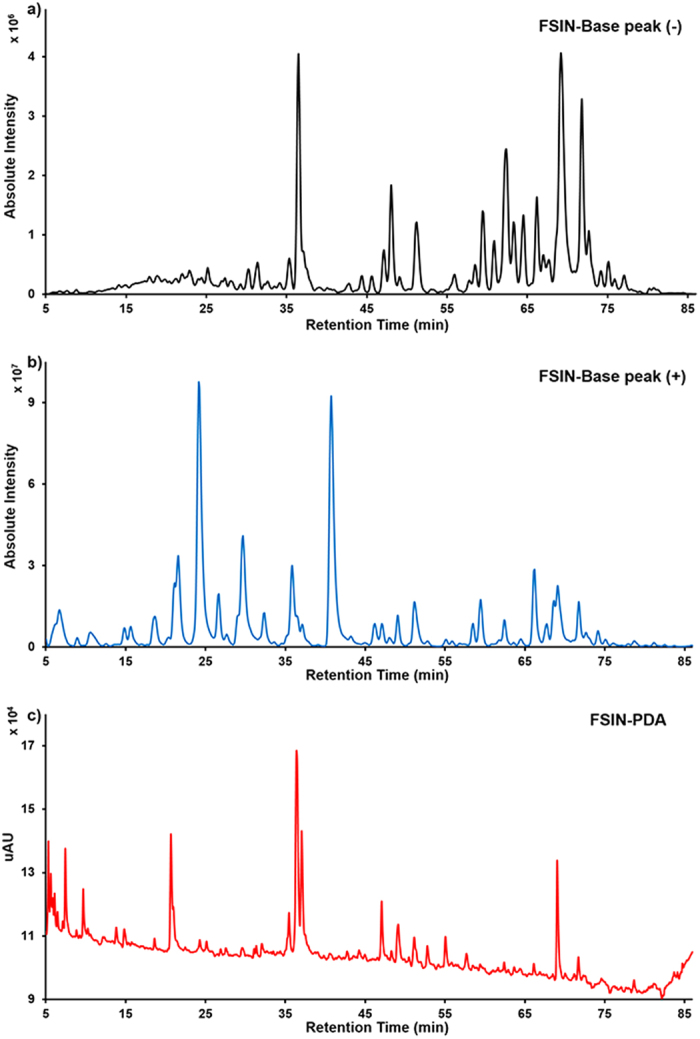
The negative base peak MS spectrum (**a**), positive base peak MS spectrum (**b**) and PDA spectrum (c) of FSIN.

Containing most of major traditional Chinese medicinal materials in SNs, FSIN was selected as the representative for identification of compounds. Multistage MS and HR-MS of FSIN were performed in both negative and positive ion modes to get complete information about its chemical constitutions. The PDA spectrum, positive base peak MS spectrum and negative base peak MS spectrum of FSIN were displayed in Fig. 2. A TCM potential target database (TCM-PTD, http://tcm.zju.edu.cn/ptd), including 12,629 ingredients related to 490 traditional Chinese medicinal materials, was regarded as an in-house database. After comparing with the reference compounds first, other molecular formulae were organized to match with those in the in-house database. Rest of compounds without matching were submitted to REAXYS database (https://www.reaxys.com/) by molecular formula. All compounds were confirmed by multistage MS data, relevant literature or UV spectra.

**Figure 3:**
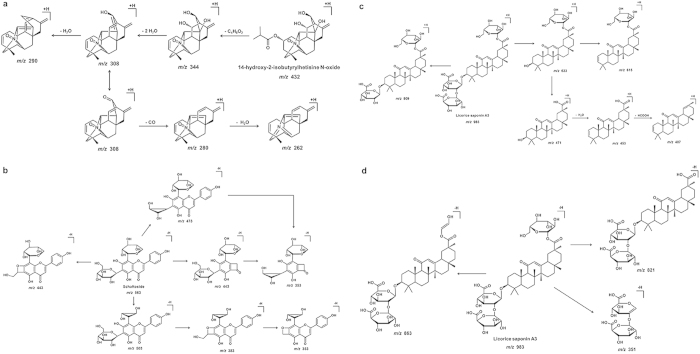
The proposed fragmentation pathways of 14-hydroxy-2-isobutyrylhetisine *N*-oxide (**a**), schaftoside (**b**) and Licorice saponin A3 (**c** and **d**).

A total of twenty alkaloids were detected in FSIN in this study. Here compound 6 at the retention time of 20.15 min was taken as an example for illustration of the identification of alkaloids as well as representative fragmentation pathways for this group of compounds. A protonated [M + H]^+^ ion peak of compound 6 was present at *m/z* 432.2362, which corresponds to elemental compositions of C_24_H_33_NO_6_. Search results in the chemical library indicated it would very likely to be 14-hydroxy-2-isobutyrylhetisine *N*-oxide. Subsequently, the MS^2^ spectrum provided a series of characteristic fragment ions for the benefit of structure determination. Fragment ions at *m/z* 414 and 344 probably be produced by the losses of H_2_O and (CH_3_)_2_CHCOOH, respectively. The existence of three consecutive fragments at *m/z* 326, 308, 290 indicated successive losses of three water molecules. In addition, characteristic ions were also detected at *m/z* 280 and 262. As shown in Fig. 3a, fragment at *m/z* 280 could be interpreted by loss of CO (−28 Da) after keto-enol tautomerism of fragment at *m/z* 308. Similarly, other alkaloids were tentatively identified using molecular formulae and characteristic fragmentation information as well as available literatures.

A total of nineteen flavonoids, including five flavones, six chalcones, seven dihydroflavones and one isoflavone, were identified as major constituents in FSIN. Compounds 19, 21, 27, 28, 32 and 41 were unambiguously identified as schaftoside, isoschaftoside, liquiritin, liquiritin apioside, isoliquiritin apioside and liquiritigenin by comparing their retention time, UV spectra and MS fragmentation with those of reference compounds. The main fragmentation mechanisms of compound 19 were first investigated to facilitate identification of other flavonoids. The proposed fragmentation pathway of compound 19 was displayed in Fig. 3b. It produced [M + H]^+^ ion at *m/z* 565.1540 and [M − H]^−^ ion at *m/z* 563.1388, which led to its molecular formula of C_26_H_28_O_14_. Along possible molecular formula inform chemical library, absorption in PDA spectrum at about 220 nm and 270 nm suggested that compound 19 was a flavonoid. Over 500 Da of molecular weight implied this likely is a conjugated flavonoid since typical molecular weight of flavonoid aglycones usually ranges from 200 to 350 Da. The retro-Diels-Alder (RDA) cleavage of C-ring bonds in the flavonoid aglycone usually generate the structural fragment at *m/z* 443 ([M − H − 120]^−^). It revealed that there was no other substituent in the B-ring. The characteristic fragments, *m/z* 473 ([M − H − 90]^−^), *m/z* 503 ([M − H − 60]^−^), and *m/z* 545 ([M − H − 18]^−^), were derived from the cleavage of sugar units or water loss from compound 19. The fragment *m/z* 383 (−180 Da) was also observed because of different combinations of the cleavage of sugar units and further loss of CH_3_OH from *m/z* 383 led to the peak with *m/z* 353. And there was no information detected due to eliminating a saccharidic residue. All the above information pointed out that compound 19 was a di-*C*-glycosyl flavonoid, but not a di-*O*-glycosyl flavonoid. In flavonoid *C*-glycosides, the sugar unit is most usually directly connected to the position 6-C or 8-C of the flavonoid aglycone. [M − H − 60]^−^ and [M − H − 90]^−^ are characteristic product ions formed by cross-ring cleavages in a pentose residue. While [M − H − 120]^−^ and [M − H − 90]^−^ are characteristic fragments formed by cross-ring cleavages in a hexose residue. Taken together, compound 19 was tentatively identified as schaftoside or isoschaftoside. Compared with reference compounds, compound 19 was undoubtedly identified as schaftoside. Based on the typical fragmentation mechanisms of schaftoside, other flavonoids were tentatively identified by molecular formulae matching, characteristic fragmentation information, UV spectra as well as the relevant literature.

Totally thirty-six triterpenoids from FSIN were identified, including nine ginsenosides. Seven of them were also unambiguously identified by comparison with the reference compounds. For example, compound 47 with a retention time of 59.39 min was detected quasi-molecular ions [M − H]^−^ at *m/z* 983 and [M + H]^+^ at *m/z* 985 in HPLC-ESI-MS^n^, and *m/z* 983.4504 in HR-MS, suggesting a molecular formula of C_48_H_72_O_21_. In the negative ion mode, a prominent ion at *m/z* 821, corresponding to the neutral loss of 162 Da from quasi-molecular ion, indicated the existence of a glucose unit. Minor ion at *m/z* 863 ([M − H − 120]^−^) from cross-ring cleavage in a saccharide residue is in agreement with the presence of a glucose unit. Characteristic ion at *m/z* 351 was also detected, which manifested that compound 47 has a GluA-GluA chain except for glucose and the GluA-GluA chain does not connect with the glucose unit. Compound 47 including a glucose unit and two glucuronic acid units can be confirmed in the positive ion mode. The [M + H]^+^ ion of compound 47 yielded an aglycone residue ion at *m/z* 471 after eliminating two glucuronic acid residues and one glucose unit consecutively. In addition, aglycone residue produced ions at *m/z* 453 and 407 by loss of a H_2_O and an extra HCOOH. This explained that the skeleton of its aglycone includes a hydroxyl group and a carboxyl group. The proposed fragmentation pathways of compound 47 were present in Fig. 3c and Fig. 3d. Compound 47 was tentatively identified as Licorice saponin A3 based on this information as well as the chemical library.

### Systematic identifications of SNs

As shown in Table 1, five SNs contain overlapping medicinal materials. The similar identification methods mentioned above were applied to major components identification in other four formulae. The overlapped medicinal material composition of five SNs sped up the comprehensive identification of chemical profile of other four formulae. The results of major components identification in five SNs were displayed in Table 2. There were altogether eighty-three compounds have been identified and their detailed mass spectrometry data were on display in the Supplementary Material (Table S1). As shown in Table 2, a compound was flagged as “√” if it existed in a formula.

### *In vivo* profiling of SNs in rat plasma

**Figure 4:**
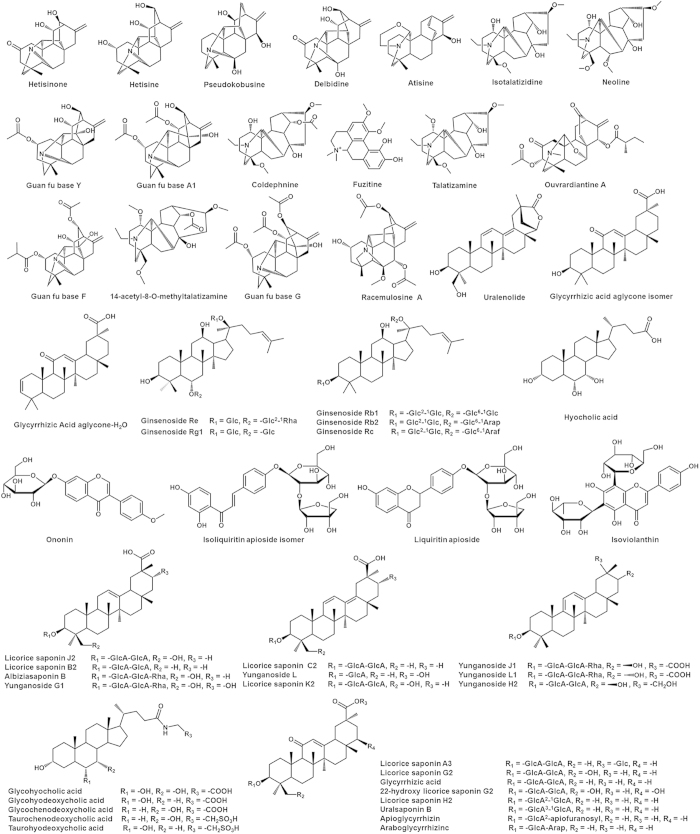
Chemical structures of prototype compounds in dosed plasma.

HPLC-ESI-MS analysis of sample plasma obtained after oral administration of five SNs was carried out both in positive and negative mode. Comparisons among the chromatograms of the blank plasma, dosed plasma, and aqueous extract in both two modes indicated that 55 compounds from aqueous extract were absorbed into the circulatory system. The 55 prototype compounds includes 18 alkaloids, 5 flavonoids, 5 ginsenosides, 21 triterpenoids and 6 bile acids. Since bile acids could be endogenous compounds in rats, variation of peak areas between chromatograms were also utilized to confirm the source of these bile acids. There are three peaks in dosed plasma displayed more than five-fold increase of peak area relative to those in blank plasma. These three peaks were designated as taurohyodeoxycholic acid, glycohyodeoxycholic acid and taurochenodeoxycholic acid, which were the major constituents of *Suis fellis pulvis* in the formula. All these absorbed components were more likely to be the bioactive components. The compounds in rat plasma were flagged with the red “√” in shadow in Table 2 and the structures of prototype compounds are shown in Fig. 4.

Biotransformations of compounds frequently produce active metabolites *in vivo*^9^. Here an approach was developed to detect metabolites by comparative analysis of HRMS data between dosed-sample and blank-sample. Three requisite criteria were applied, which are accuracy mass of precursor ions within an error of 5 ppm, isotope ratio tolerance less than 10 and fold change of intensity more than 5. The metabolites are divided into two groups based on whether there is effective MS^2^ fragment ions. A metabolite belongs to group 1 (G1) if relevant MS^2^ fragment ions can give evidence of it. Otherwise, a metabolite belongs to group 2 (G2). Metabolites profiling of SNs in rat plasma are shown in Table 3. There are a total of thirty-nine metabolites detected in SNs and some of their MS^2^ data are displayed in the Table S2 (Supplementary Material).

## Discussion

This is the first report describing the difference of chemical compositions among five SNs and Table 1 summarized the composition of these analogous formulae. This group of analogous formulae mainly consists of *Aconiti lateralis radix, Glycyrrhizae radix et rhizome praeparata cum melle* and *Zingiberis rhizoma* with different weight ratio or adding *Suis feellis pulvis* or *Ginseng radix et rhizoma* and/or *Poria*. Negative and positive base peak mass spectrums were measured for five SNs to obtain complete information about their chemical constituents. Consisting of majority of TCM materials in SNs, FSIN was selected as the representative of SNs and qualitative identification study was performed on this formula first to establish a basic chemical profile. With availability of composition of medicinal material for all five SNs, peaks in other four SNs were first allocated to previously identified compounds in FSIN when two formulae shared same TCM material. The unique compounds in each formula were then identified separately. This workflow significantly facilitated the identification process under preliminary requirement on identical experimental conditions for all five SNs that made their chromatograms are directly comparable.

As shown in results, alkaloids, flavonoids and triterpenoids comprised of majority of compounds in SNs. Alkaloids are the major bioactive components from *Aconiti lateralis radix*. The most significant characteristics of these alkaloids is the presence of a relatively high-intensity protonated molecular ion [M + H]^+^ in their positive-ion spectrum while almost no response in negative-ion mode. For flavonoids, [M − H]^−^, [2M − H]^−^, [M + HCOOH − H]^−^ are the major high-intensity ions in the negative mode. These ions are conducive to confirming their molecular weights and molecular formulae. In ESI-MS^n^ spectra, the main fragment ions of alkaloids are originated from the neutral loss of 18 Da, 32 Da, and 60 Da or their superposition in accordance to loss of a molecule of water, methanol, acetic acid or a combination of them, respectively. The relative abundance of these fragments not only depend on the nature but also is sensitive to the position of the substituents on the rings^20^. The fragmentation behaviors of alkaloids observed in this study were very consistent with previously reports^21^,^22^. While flavonoid glycosides in ESI-MS^n^ spectra, were characterized by loss of 162 Da, 132 Da or 146 Da on account of the elimination of hexose, pentose or rhamnose, respectively. Compared with alkaloids and flavonoids, triterpenoids have relatively higher molecular weight and smaller polarity. Triterpenoids, including ginsenosides and licorice saponin compounds, comes mainly from *Ginseng radix et rhizoma* and *Glycyrrhizae radix et rhizome praeparata cum melle*. Ginsenosides generally yield singly charged ions [M − H]^−^ and [M + HCOOH−H]^−^ in negative ion mode, availing the determination of molecular formula. Characteristic ions [aglycone−H]^−^ at *m/z* 475 or 459 after loss of all glycosidic units can be observed in the MS/MS spectra, which could be used to discriminate protopanaxatriol from protopanaxadiol type ginsenosides. In addition, MS/MS spectra suggested the amount and species of sugar moieties and a mass difference of 162, 132 or 146 indicated the presence of a glucose, pentose or rhamnose moiety, respectively. Licorice saponin compounds are the major active compounds in *Glycyrrhizae radix et rhizome praeparata cum melle* and exhibit high abundances of quasi-molecular ions [M − H]^−^. For licorice saponin compounds, neutral loss of 176 Da, 162 Da or 146 Da from [M − H]^−^ also heralds that the relevant compounds possess a glucuronic acid (GluA) unit, a glucose unit or a rhamnose (Rham) unit. Furthermore, predominant saccharide chain ions, such as *m/z* 351, 497 or 339, are remarkably characteristics in licorice saponin compounds, signifying the relevant compounds with a GluA-GluA chain, a GluA-GluA-Rham chain or a GluA-Rham chain.

In general, many nature products from plants have similar structures or skeletons. Like flavonoids or triterpenoids, a number of them shared a common basic skeleton with only minor differences on substituents. Polarity and molecular weight determines their retention time in LC. In different MS spectra, the fragments information from multiage MS of every peak can help determine whether two peaks within 1 min changes of retention time were the same compound or not. The overlapped medicinal material composition of five SNs sped up the comprehensive identification of chemical profile of other four formulae.

It is widely assumed that non-absorbed compounds are most unlikely to become active constituent after oral administration of TCM, except for compounds directly exerting effects on the gastrointestinal tract^11^,^23^. Hence, it is very meaningful to confirm which compounds were absorbed into blood by rats after oral administration of SNs. This effort provides useful information to screen active ingredients and imply the different action mechanisms among analogous formulae. 55 prototype compounds, including 18 alkaloids, 5 flavonoids, 5 ginsenosides, 21 triterpenoids and 6 bile acids from aqueous extract absorbed into the circulatory system were extracted. Alkaloids from *Aconiti lateralis radix* were the major group of compounds for these five SNs *in vivo*, which are known for many pharmacological effects, such as cardiotonic effect, anti-inflammatory, antioxidant^24^,^25^,^26^,^27^. Studies showed that alkaloids have protective effect on cardiovascular system, mainly reflecting in improving heart function, stabilizing blood pressure, antagonizing coagulation and thrombosis and easing ischemia reperfusion injury^28^,^29^,^30^. Flavonoids and triterpenoids are bioactive compounds presented in *Glycyrrhizae radix et rhizome praeparata cum melle* and exhibit diverse biological effects^31^,^32^,^33^,^34^,^35^. Both experimental and clinical studies suggested that they possess cardioprotective, antioxidative, hepatoprotective properties^36^. Bile acids were unique components in ZSIN, which come from *Suis fellis pulvis*, one of the most commonly used traditional medicines in China^37^,^38^. Modern pharmacological research suggested that they displayed diverse biological activities, such as anti-inflammatory effect, treating gallstones, hypolipidemic effect^39^,^40^. For example, taurohyodeoxycholic acid has protective effect on ulcerative colitis in mice, which might be partially due to suppressing pro-inflammatory mediators TNF-α and IL-6^39^. Previous work showed that absorbed taurochenodeoxycholic acid could exert a good anti-adjuvant arthritis activity in rats by directly inhibiting the activity of NF-*κ*B and reducing expression of diverse inflammatory and immune response mediators^41^. Inflammation is known to an important pathophysiological factor in mediating and exacerbating cardiovascular diseases^42^,^43^ and abundant experimental data proved that anti-inflammatory action plays an important role in the prevention and treatment of cardiovascular diseases^44^,^45^,^46^. Therefore, bile acids may exhibits cardiovascular protection effects because of their anti-inflammatory activity and could be important active components responsible for therapeutic effect of ZSIN. Ginsenosides, the major constituents of *Ginseng radix et rhizoma*, were only present in RSIN and FSIN. Depending on physico-chemical properties, some of them are orally active through penetrating GI lumen to enter the systemic circulation^47^. Many researches showed that *Ginseng radix et rhizoma* and ginsenosides exerted satisfactory therapeutic effects, especially in cardiovascular disorders and neurodegenerative disorders^48^,^49^,^50^. Studies have shown that ginsenoside Rb1 protects against myocardial ischemia-reperfusion injury partially through regulating the activation of PI3K-Akt signaling pathway and attenuation of cardiac hypertrophy by inhibiting Ca^2+^−CaN signal transduction pathway^51^,^52^. Literature survey revealed that diverse biological processes could be modulated by these compounds identified *in vivo* that shed some light on mechanisms of actions of SNs in treating cardiovascular diseases. In addition to the prototype compounds, thirty-nine metabolites from biotransformation were screened. The bioactive metabolites are also important for the explanation of pharmacological efficacies of TCM formulae. To fully understand the fundamental differences between SNs from different levels, there is much work need to be done, e.g., to quantify absorbed compounds and investigate how absorbed compounds regulate biological processes.

In this study, multistage MS experiments and HR-MS experiments were performed to present chemical profiling differentiation of five SNs *in vitro* and *in vivo*. It is significant to characterize and compare the chemical constituents of a category of analogous formulae simultaneously because it will lay a solid foundation for further comparative study of their pharmacological efficacies and molecular mechanisms. Besides, comparative analysis on analogous formulae would be more efficient and effective compared with study on one single TCM formula. As shown in Table 2, 83 compounds including alkaloids, flavonoids, ginsenosides, bile acids and triterpenoids have been separated and identified by reference compounds comparison through several sources, including molecular formulae matching, characteristic fragmentation information, UV spectra as well as the relevant literature. A total of 94 compounds including 55 prototype compounds and 39 metabolites were detected in rat plasma after oral administration of formulae, which would most likely be responsible for pharmacological effects of these formulae. In summary, differential analyses of constituents in five SNs *in vitro* and *in vivo* were carried out, which was helpful for further comparative study of pharmacological and clinical studies of SNs.

## Methods

### Chemicals and materials

All herbs were purchased from Zhejiang Chinese Medical University medical pieces (Hangzhou, China). *Suis fellis pulvis* was of analytical grade and was obtained from YIJI fundamental industrial Co., LTD. (Shanghai, China). All materials were identified by Prof. Liurong Chen.

Authentic standards of ginsenoside Rg1, ginsenoside Re, ginsenoside Rf, ginsenoside Rb2, ginsenoside Rc, ginsenoside Rb1, liquiritin, glycyrrhizic acid, isoliquiritin apioside, liquiritin apioside were purchased from Shanghai Winherb Medical Technology (Shanghai, China). Liquiritigenin was obtained from Jincefenxi Technology (Tianjin, China). Schaftoside and isoschaftoside were supplied by Chengdu Must Biotechnology (Chengdu, China) and Zhongxin Innova Laboratories (Tanjin, China), respectively. Purity of all the reference substances was more than 98%.

HPLC grade acetonitrile and methanol were from Merck (Darmstadt, Germany). HPLC grade formic acid was from ROE Scientific Inc. (Newark, DE, USA). All other reagents were of analytical grade.

Preparation of standard solutions and samples. Stock solutions of the 13 reference substances were prepared by dissolving them in 25% (*v/v*) methanol through ultrasound and centrifuging at 10000 rpm for 10 min.

Analogous formulae include SIN, FSIN, RSIN, TSIN and ZSIN. The compositions of these five formulae were shown in Table 1. Decoctions were prepared according to ancient Zhang Zhongjing’s herbal formulae. The herbal pieces of each decoction were immersed in pure water (six times of total weight of dried herbs) for overnight and then heated under reflux extracted twice for 1.5 hours with six and four times of total weight of dried herbs, respectively. Extracted solutions were combined after filtered through two layers of gauze. The filtrate was evaporated to semi-dryness under reduced pressure with a rotary evaporator at 70 °C and was totally dried by vacuum freeze-drying. Samples for HPLC-MS analysis were prepared in water at the concentrations of 5 mg/mL. The samples were centrifuged at 10000 rpm for 10 min before analysis.

### High performance liquid chromatography

A Zorbax Eclipse XDB-C_18_ column (5 μm, 4.6 × 250 mm; Agilent Technologies, Santa Clara, CA, USA) was used for all the chromatographic analysis. The mobile phase consisted of 0.05% formic acid-water (*v/v*) (A) and 0.05% formic acid-acetonitrile (B), using a gradient elution of 5–30% B at 0–50 min, 30–50% B at 50–75 min, 50–95% B at 75–80 min and 95% B at 80–90 min. The sample volume injected was set at 20 μL. The column temperature was set at 30 °C. The flow rate was 0.6 mL/min and the photodiode array (PDA) detection range was from 190 to 400 nm.

### Mass spectrometry

The multistage MS data were acquired on an Agilent 1100 series HPLC system (Agilent Technologies, Waldbronn, Germany) coupled with an LCQ Deca XP^plus^ ion trap mass spectrometer (IT-MS) (Thermo Finnigan, San Jose, CA, USA) via a commercial ESI interface. The instrument settings of multistage MS experiments were as follows: the scan mass range was set at *m/z* 100–1500 in both positive and negative ion mode with +4.00 kV and −3.00 kV source voltage, respectively. The capillary temperature was set at 350 °C, with sheath gas N_2_ pressure 60 arb and auxiliary gas N_2_ pressure 20 arb.

The HR-MS analyses were performed with Waters UPLC (Waters Corp., Milford, MA, USA) equipped with an AB SCIEX Triple TOF 5600^plus^ System (AB SCIEX, Framingham, MA, USA). The TOF-MS analysis was performed using in both positive and negative ion mode with *m/z* 100–1500. The conditions of ESI source were as follows: CUR, 30 psi; GS1, 50 psi; GS2, 50 psi; ISVF, −4.5 kV or +5.5 kV; TEM, 550 °C for negative ion mode and 600 °C for positive ion mode. Injection volume for aqueous extracts was set at 10 μL. A margin of error up to ±5 ppm was allowed. Both IT-MS and HR-MS data were acquired during 5–90 min with the same liquid chromatography conditions described above.

### HPLC-MS chemical profiling method validation

The precision of the HPLC-MS method was determined by the intra- and inter-day variations and expressed as percentage relative standard deviation (RSD) of replicate. In practical terms, one sample was analyzed for six times within a day to test the intra-day precision, while one sample was tested in duplicates for consecutive three days to evaluate the inter-day precision. To evaluate the repeatability, six replicates of the same sample were prepared and analyzed. The stability was assessed by analyzing the same sample storing at room temperature at 0, 2, 4, 8, 16 and 24 h. The RSDs of replicate were applied to evaluating the repeatability and stability.

### Animal experiments

Twenty-five male Sprague-Dawley (SD) rats were purchased from Shanghai SLAC laboratory Animal Co., Ltd. (Shanghai, China). The rats were acclimated for a week with temperature of 24 ± 2 °C, humidity of 55 ± 15%, and 12 h dark-light cycle. Before the experiments, all rats were fasted overnight with free access to water. Twenty-five male SD rats, weighing between 200 ± 10 g, were randomly separated into 5 groups and administered once with 8 times daily dosage of aqueous extracts via gastric gavage (the detailed information of oral single-dose of SNs in Table 4 and 3.2 mL per 100 g body weight). About 1 mL blood samples were collected from eyes in heparinized tubes immediately before dosing and at 60 min after dosing, respectively. The plasma was separated after blood samples centrifuging at 4000 rpm for 15 min and was stored at −80 °C for later analysis.

All procedures about animal care and experiments were performed in accordance with protocols which were approved by the Animal Ethic Review Committees of Zhejiang University.

### Preparation of plasma samples

To eliminate the individual variability among the animals, plasma samples of post-dosed rats in the same group were mixed together as one dosed sample for each formula and twenty-five plasma samples from pre-dosed rats were combined into one blank sample. Each 0.5 mL aliquot of plasma sample was mixed with 1 mL of acetonitrile and vortexes for protein precipitation. After centrifugation at 10000 rpm for 10 min, the supernatant was transferred and concentrated to dryness at 35 °C. The dried residue was then re-dissolved in 100 μL of methanol by ultrasound. After centrifugation again at 10000 rpm for 10 min, the supernatant was transferred to auto-sampler vial. Injection-volumes were 30 μL and 7.5 μL for HPLC-MS and UPLC-HRMS analysis, respectively.

### Methods of data processing

A TCM potential target database (TCM-PTD, http://tcm.zju.edu.cn/ptd) developed internally was regarded as an in-house database to identify chemical component in SNs. A total of 12,629 ingredients related to 490 traditional Chinese medicinal materials covering Chinese Pharmacopeia 2010 Edition (Volume I) were collected in current TCM-PTD (Version 1.0). All respects of traditional Chinese medicinal materials including name, ingredient, molecular formula, molecular weight and 2D structure were collected. After comparing with the reference compounds, other molecular formulae were organized to match the in-house database. Those compounds without match in the TCM-PTD were retrieved on the REAXYS database (https://www.reaxys.com/), which covers fields of chemical research in academia and industry. Possible hits from the REAXYS database were then narrowed by filtering with origin of the compounds to natural product and/or substructure. All compounds were confirmed by multistage MS data, relevant literature and/or UV spectra.

The prototype compounds absorbed into the circulatory system were detected by comparisons among the chromatograms of the blank plasma, dosed plasma, and aqueous extract in both two modes.

PeakView™ software v1.2 with the XIC Manager add-in was used for finding potential metabolites by HRMS data comparative analysis between dosed-sample and blank-sample. This approach covers 4 steps. Firstly, the possible metabolic pathways of a parent compound were listed^53^ (shown in Table 5). Secondly, Theoretical metabolites of each compound in SNs were generated quickly by Python script program based on the mainly metabolic pathways. Thirdly, potential metabolites were screened with high confidence based on accurate mass molecular ion, isotopic pattern, nitrogen rule and the ratio of the dosed-sample intensity to the blank-sample intensity (fold change of intensity). Forth, metabolites were confirmed through analyzing their MS^2^ data and comparing with the prototype.

## Additional Information

**How to cite this article**: Chen, Q. *et al*. Chemical and Metabolic Profiling of Si-Ni Decoction Analogous Formulae by High performance Liquid Chromatography-Mass Spectrometry. *Sci. Rep*. **5**, 11638; doi: 10.1038/srep11638 (2015).

## Supplementary Material

Supplementary Information

## Figures and Tables

**Figure 1:**
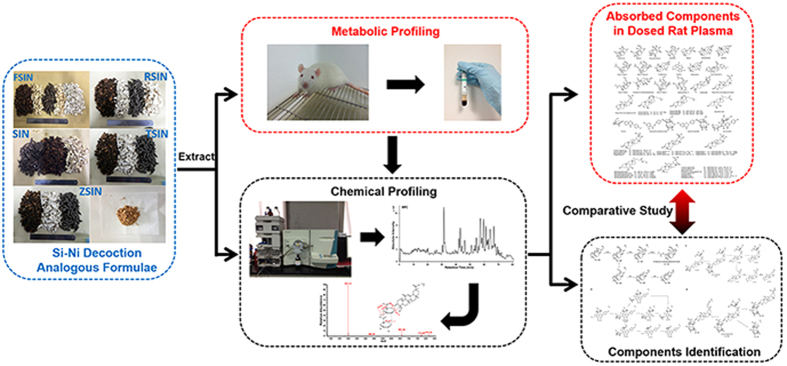
Comparative analysis scheme of Si-Ni decoction analogous formulae *in vitro* and *in vivo*.

**Table 1:** Composition of Si-Ni Decoction Analogous Formulae.

Traditional Chinese Medicinal Materials (g)	Si-Ni Decoction Analogous Formulae				
	SIN	RSIN	TSIN	ZSIN	FSIN
*Glycyrrhizae radix et rhizome praeparata cum melle*	31.2	31.2	31.2	31.2	15.6
*Zingiberis rhizoma*	23.4	23.4	46.8	46.8	11.7
*Aconiti lateralis radix*	25	25	30	30	12.5
*Ginseng radix et rhizoma*		15.6			7.8
*Poria*					31.2
*Suis fellis pulvis*				6	

**Table 2:**
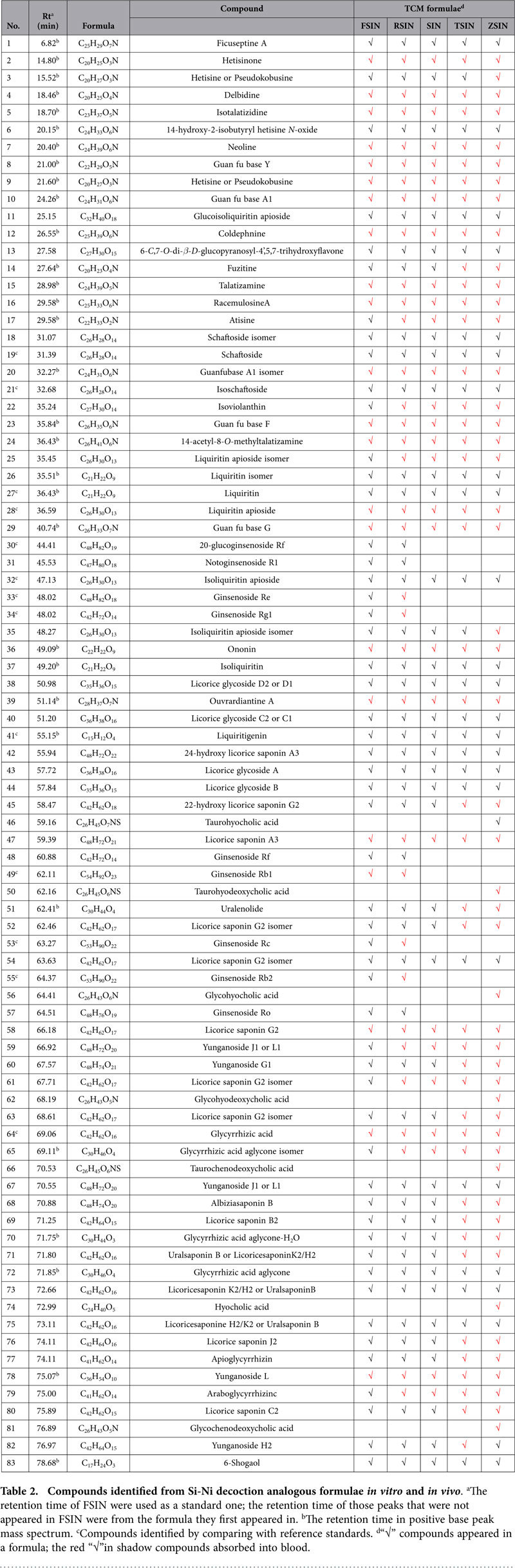
Compounds identified from Si-Ni decoction analogous formulae *in vitro* and *in vivo*.

**Table 3:** Metabolic profiling of Si-Ni decoction analogous formulae in rat plasma.

No.	Rt^a^ (min)	Formula	Metabolic pathway	Error (ppm)	Prototype	Group	Fold change of intensity
M1	14.26^b^	C_22_H_35_O_5_N	Demethylation	0.7	Isotalatizidine	G1	15.10
			Deethylation		Talatizamine		
M2	15.11^b^	C_20_H_23_O_3_N	Alcohol to ketone	0.2	Hetisinone	G2	5.70
			Loss of O		Fuzitine		
			Alcohols dehydration		Delbidine		
M3	18.15^b^	C_22_H_31_O_6_N	Hydrolysis	0.3	Guan fu base Y	G1	7.73
M4	19.75^b^	C_24_H_37_O_6_N	Hydroxylation + desaturation	0.9	Talatizamine	G1	8.67
			Alcohol to ketone		Neoline		
			Demethylation		Coldephnine		
			Deethylation		14-acetyl-8-*O*-methyltalatizamine		
M5	28.94^b^	C_26_H_33_O_8_N	Hydroxylation	0.5	Guan fu base G	G1	25.48
M6	29.29^b^	C_26_H_33_O_9_N	2 × hydroxylation	−0.1	Guan fu base G	G1	5.12
M7	29.57^b^	C_25_H_37_O_6_N	Alcohol to ketone	0.8	Coldephnine	G2	13.46
M8	33.30^b^	C_25_H_39_O_5_N	Loss of O	−0.8	Coldephnine	G1	5.09
			Hydroxymethylene loss		14-acetyl-8-*O*-methyltalatizamine		
M9	34.99	C_15_H_10_O_5_	Hydroxylation + desaturation	0.4	Liquiritigenin	G2	30.23
M10	39.64^b^	C_22_H_20_O_11_	Demethylation to carboxylic acid	0.5	Ononin	G1	9.48
M11	40.63^b^	C_28_H_37_O_8_N	Hydroxylation	0.3	Ouvrardiantine A	G1	16.77
M12	41.18	C_26_H_30_O_12_	Loss of O	4.9	Liquiritin apioside isomer	G1	34.68
			Loss of O		Liquiritin apioside		
			Loss of O		Isoliquiritin apioside		
			Loss of O		Isoliquiritin apioside isomer		
M13	42.06^b^	C_26_H_33_O_6_N	Alcohol to ketone	−0.1	Guan fu base F	G1	8.78
			Loss of O		Guan fu base G		
M14	42.20^b^	C_15_H_12_O_5_	Hydroxylation	0.8	Liquiritigenin	G1	4.98
M15	42.20^b^	C_21_H_20_O_11_	Hydroxylation + glucuronide conjugation	0.2	Liquiritigenin	G1	43.90
			Demethylation + 2 × hydroxylation		Ononin		
M16	42.55^b^	C_22_H_22_O_11_	2 × hydroxylation	0.4	Ononin	G1	83.02
M17	42.56^b^	C_16_H_14_O_5_	Hydroxylation +methylation	0.7	Liquiritigenin	G1	27.74
M18	43.27^b^	C_27_H_35_O_7_N	Demethylation	0.8	Ouvrardiantine A	G1	11.40
			Methylation		Guan fu base G		
			Acetylation		Racemulosine A		
M19	44.36	C_15_H_12_O_7_S	Sulfate conjugation	1.0	Liquiritigenin	G2	21.13
M20	47.13^b^	C_22_H_20_O_10_	Hydroxylation + desaturation	0.5	Ononin	G1	13.79
M21	49.07^b^	C_21_H_20_O_10_	Hydroxylation + desaturation	0.7	Liquiritin isomer	G1	80.87
			Hydroxylation + desaturation		Liquiritin		
			Hydroxylation + desaturation		Isoliquiritin		
			Glucuronide conjugation		Liquiritigenin		
M22	50.71^b^	C_15_H_10_O_4_	Alcohol to ketone	0.4	Liquiritigenin	G1	8.22
M23	50.79	C_17_H_14_O_5_	Acetylation	0.8	Liquiritigenin	G2	7.72
M24	54.90^b^	C_34_H_42_O_19_	Acetylation	4.3	Glucoisoliquiritin apioside	G2	33.37
M25	55.67^b^	C_28_H_37_O_12_N	Hydroxylation + glucuronide conjugation	4.7	Guan fu base Y	G1	7.54
M26	55.95	C_22_H_22_O_10_	hydroxylation	−0.8	Ononin	G1	22.37
M27	56.00^b^	C_16_H_14_O_4_	Methylation	0.6	Liquiritigenin	G1	10.21
M28	56.42	C_32_H_51_O_11_N	Glucuronide conjugation	−0.9	Glycohyodeoxycholic acid	G2	15.58
			Glucuronide conjugation		Glycochenodeoxycholic acid		
M29	65.44^b^	C_26_H_41_O_4_N	Alcohols dehydration	0.6	Glycohyodeoxycholic acid	G2	11.65
			Alcohols dehydration		Glycochenodeoxycholic acid		
M30	69.09^b^	C_26_H_41_O_5_N	Alcohol to ketone	0.8	Glycohyodeoxycholic acid	G1	8.82
			Alcohol to ketone		Glycochenodeoxycholic acid		
			Alcohols dehydration		Glycohyocholic acid		
M31	70.91	C_50_H_77_O_22_N	Glycine conjugation	2.9	Yunganoside G1	G2	8.21
			Taurine conjugation		Yunganoside J1 or L1		
M32	72.90	C_23_H_32_O_10_	Hydroxylation + glucuronide conjugation	−2.2	6-Shogaol	G2	7.92
M33	74.64	C_31_H_47_O_13_N	Hydroxylation + glucuronide conjugation	0.4	Coldephnine	G2	11.76
M34	75.56	C_26_H_43_O_5_NS	Alcohols dehydration	−0.9	Taurohyodeoxycholic acid	G1	17.01
			Alcohols dehydration		Taurochenodeoxycholic acid		
M35	76.37	C_28_H_45_O_6_N	Acetylation	−0.6	Glycohyodeoxycholic acid	G2	12.57
			Acetylation		Glycochenodeoxycholic acid		
M36	80.29	C_30_H_48_O_5_	Hydrolysis	−3.3	Glycyrrhizic acid aglycone isomer	G2	5.04
			Hydrolysis		Glycyrrhizic acid aglycone		
M37	80.43	C_31_H_46_O_5_	Hydroxylation + methylation	−0.8	Uralenolide	G2	6.87
M38	81.14	C_31_H_48_O_5_	Hydroxylation + methylation	0.7	Glycyrrhizic acid aglycone isomer	G2	6.68
			Hydroxylation + methylation		Glycyrrhizic acid aglycone		
M39	83.58	C_31_H_48_O_4_	Methylation	−2.2	Glycyrrhizic acid aglycone isomer	G2	12.38
			Methylation		Glycyrrhizic acid aglycone		

^a^The retention time of FSIN were used as a standard one; the retention time of those peaks that were not appeared in FSIN were from the ZSIN.

^b^The retention time in positive base peak of high resolution mass spectrum.

**Table 4:** The oral single-dose of SNs treated to rats for analysis *in vivo*.

Si-Ni Decoction Analogous Formulae	Daily Dosage^a^ (g/kg)	Oral Single-dose^b^ (g/kg)
SIN	7.2	57.3
RSIN	8.6	68.5
TSIN	9.7	77.8
ZSIN	10.6	85.0
FSIN	7.1	56.7

^a^Daily dosage means the dosage for per kilogram body weight rat every day. It was translated from a human equivalent dose taking into account body weight and body surface area.

^b^Oral single-dose is the actual dosage in our animal experiment. Based on the maximal oral dosage and the solubility of SNs, the oral single-dose of SNs was 3.2 mL per 100 g body weight (i.e. 8 times daily dosage).

**Table 5:** The mainly metabolism pathway of xenobiotics and the formula change relative to the parent compound.

Metabolic pathway	Formula change of the parent compound	Metabolic pathway	Formula change of the parent compound
Decarboxylation	–COOH	Demethylation + hydroxylation	−CH_2_ + O
Hydrolysis	+H_2_O	Deethylation	−C_2_H_4_
Hydroxylation	+O	Isopropyl dealkylation	−C_3_H_6_
Demethylation + 2 × hydroxylation	−CH_2_ + O_2_	Methylation	+CH_2_
Reduction	+H_2_	Acetylation	+C_2_H_2_O
Hydroxylation + desaturation	+O−H_2_	S-cysteine conjugation	+C_3_H_5_NO_2_S
Alcohol to ketone	−H_2_	Glycine conjugation	+C_2_H_3_NO
Demethylation	−CH_2_	Taurine conjugation	+C_2_H_5_NO_2_S
Hydroxymethylene loss	−CH_2_O	Cysteine conjugation	+C_3_H_5_NOS
Loss of O	−O	Glucuronide conjugation	+C_6_H_8_O_6_
2 × hydroxylation	+O_2_	Hydroxylation + glucuronide conjugation	+C_6_H_8_O_7_
Demethylation to carboxylic acid	−CH_2_ + CO_2_	Sulfate conjugation	+SO_3_
Alcohols dehydration	−H_2_O	Hydroxylation + sulfation	+SO_4_
Hydroxylation + methylation	+O + CH_2_	Decarboxylation + glucuronidation	−CO + C_6_H_8_O_6_
