# Supplementary material for: Chemical and Metabolic Profiling of Si-Ni Decoction Analogous Formulae by High performance Liquid Chromatography-Mass Spectrometry
Source: Sci Rep. 2015 Jun 29;5:11638. doi: 10.1038/srep11638 (PMC4484491; doi:10.1038/srep11638)
Supplement: Supplementary Information [file srep11638-s1.doc]

***Supplementary Material for:***

Chemical and Metabolic Profiling of Si-Ni Decoction Analogous Formulae by High performance Liquid Chromatography-Mass Spectrometry

*Qian Chen, Shun Xiao, Zhenhao Li, Ni Ai and Xiaohui Fan**

*Pharmaceutical Informatics Institute, College of Pharmaceutical Sciences, Zhejiang University, Hangzhou 310058, China; E-Mail: fanxh@zju.edu.cn*

** Author to whom correspondence should be addressed; E-Mail: fanxh@zju.edu.cn;
Tel.: +86-571-88208596; Fax: +86-571-88208426.*

**Table S1.** Detailed MS data of FSIN.

| **Rt**  **（min）** | **MS Neg** | **MS Pos** | **MS^2^ Neg** | **MS^2^ Pos** | **MW (HR-MS)** | **Formula** | **Error (ppm)** |  |
| --- | --- | --- | --- | --- | --- | --- | --- | --- |
| **6.82^a^** |  | MS[Full ms]457.08(87), 456.04(510), 438.18(42), 409.15(21), 378.16(21), 344.09(26), 300.19(43), 296.06(26), 295.14(150), 294.11(1000), 277.26(27), 276.29(215), 258.25(45), 230.39(31), 132.05(85) |  | MS2[456.00@35]: 437.98(1000), 420.13(18), 410.14(14), 276.11(56), 258.20(8) | 456.2039 | **C_25_H_29_O_7_N** | 4.9 |  |
| **14.80^a^** |  | MS[Full ms]417.08(20), 393.26(17), 392.26(22), 391.25(18), 362.15(18), 330.41(28), 329.34(234), 328.34(1000), 284.17(20) |  | MS2[328.30@35]: 329.27(253), 328.26(1000), 311.35(13), 310.36(154), 300.39(6), 292.38(7) | 328.1897 | **C_20_H_25_O_3_N** | -3.1 |  |
| **15.52^a^** |  | MS[Full ms]529.22(64), 373.17(48), 332.46(32), 331.39(241), 330.39(1000), 329.73(47), 328.51(46), 312.53(66) |  | MS2[330.40@35]: 331.23(120), 330.23(362), 329.41(13), 313.16(222), 312.19(1000), 295.32(101), 294.31(557), 293.70(15) | 330.2056 | **C_20_H_27_O_3_N** | -2.3 |  |
| **18.46^a^** |  | MS[Full ms]900.13(11), 899.12(16), 370.36(36), 362.20(19), 346.36(38), 345.30(240), 344.31(1000), 122.23(12), 109.90(14) |  | MS2[344.30@35]: 345.20(305), 344.23(1000), 343.47(11), 327.24(82), 326.25(611), 309.29(16), 308.25(112), 280.30(17), 267.10(13), 263.28(16), 211.12(12) | 344.1845 | **C_20_H_25_O_4_N** | -3.3 |  |
| **18.70^a^** |  | MS[Full ms]410.35(42), 409.37(242), 408.38(1000), 407.75(23), 390.59(48), 347.68(50), 346.52(226), 345.64(34), 344.55(233), 250.39(33) |  | MS2[408.40@35]: 409.21(202), 408.22(367), 391.23(225), 390.28(1000), 389.68(44), 372.30(66), 358.43(17) | 408.2728 | **C_23_H_37_O_5_N** | -4.0 |  |
| **20.15^a^** |  | MS[Full ms]504.34(64), 434.30(62), 433.37(217), 432.31(1000), 408.45(83), 388.54(51), 378.54(51), 360.44(121), 314.42(224), 122.49(64), 110.08(84) |  | MS2[432.30@35]: 433.30(57), 432.26(108), 415.15(78), 414.14(302), 350.29(69), 344.16(232), 327.14(123), 326.11(642), 309.12(208), 308.09(1000), 291.11(139), 290.08(679), 280.15(271), 263.11(163), 262.19(290), 260.20(54), 245.17(283), 235.13(114), 233.05(84) | 432.2362 | **C_24_H_33_O_6_N** | -4.3 |  |
| **20.40^a^** |  | MS[Full ms]439.44(107), 438.35(473), 432.30(65), 408.49(36), 390.27(51), 389.34(244), 388.36(1000), 387.54(41), 344.49(36), 328.45(33), 314.40(32), 266.29(43), 265.25(275) |  | MS2[438.40@35]: 439.36(6), 438.27(13), 407.32(200), 406.30(1000), 389.34(6), 388.38(75), 374.45(10), 370.25(6), 356.30(17), 347.76(6), 344.17(7), 313.22(6) | 438.2850 | **C_24_H_39_O_6_N** | -4.4 |  |
| **21.00^a^** |  | MS[Full ms]661.57(9), 645.28(8), 620.95(8), 391.37(8), 390.38(31), 389.33(229), 388.36(1000), 352.63(7), 351.94(6), 292.41(7), 195.18(13) |  | MS2[388.40@35]: 389.17(426), 388.15(789), 370.03(83), 353.01(243), 352.00(1000), 329.19(187), 328.15(883), 324.14(165), 310.25(105), 293.21(141), 292.16(893), 144.13(119) | 388.2105 | **C_22_H_29_O_5_N** | -3.5 |  |
| **21.60^a^** |  | MS[Full ms]388.26(39), 332.33(26), 331.34(215), 330.36(1000) |  | MS2[330.40@35]: 331.25(249), 330.30(1000), 312.56(60), 294.43(9) | 330.2057 | **C_20_H_27_O_3_N** | -2.0 |  |
| **24.26^a^** |  | MS[Full ms]433.13(15), 432.25(42), 431.27(228), 430.31(1000), 370.70(11) |  | MS2[430.30@35]: 431.17(92), 430.16(187), 412.12(33), 371.20(203), 370.20(1000), 353.14(55), 352.16(305), 342.26(36), 324.21(82), 311.25(44), 310.26(251), 293.28(35), 292.25(186), 264.33(38), 144.19(26) | 430.2208 | **C_24_H_31_O_6_N** | -3.8 |  |
| **25.15** | MS[Full ms]1455.30(68), 1429.68(229), 1340.83(88), 1299.41(150), 1290.59(132), 1166.40(504), 1067.25(32), 824.89(57), 810.91(47), 759.05(77), 758.08(251), 756.94(1000), 755.88(37), 755.26(39), 749.23(55), 747.22(112), 712.15(79), 711.19(367), 549.46(52) |  | MS2[711.20@35]: 693.24(2), 666.51(3), 650.94(3), 643.48(3), 632.00(4), 620.01(5), 549.24(1000), 429.11(3) |  | 711.2119 | **C_32_H_40_O_18_** | -3.2 |  |
| **26.55^a^** |  | MS[Full ms]452.31(46), 451.36(211), 450.38(1000), 449.68(16), 433.00(21), 432.34(26), 431.53(13), 430.56(32), 110.01(14) |  | MS2[450.40@35]: 451.21(189), 450.19(274), 433.22(191), 432.26(1000), 414.27(51), 400.38(11), 354.08(14), 340.18(12), 322.11(11) | 450.2836 | **C_25_H_39_O_6_N** | -3.1 |  |
| **27.58** | MS[Full ms]1495.83(87), 1360.84(108), 1329.31(95), 1304.34(82), 1289.90(111), 1259.28(101), 1242.83(686), 1144.37(87), 1124.41(298), 1041.42(89), 1037.75(86), 1019.03(95), 920.56(91), 900.99(90), 800.33(82), 599.74(105), 595.32(103), 594.27(241), 593.37(1000) | MS[Full ms]596.02(64), 595.06(221), 451.41(53), 450.30(153), 447.27(57), 446.22(174), 430.30(126), 414.37(74), 374.33(111), 343.19(197), 342.21(1000), 297.23(91), 109.99(58) | MS2[593.40@35]: 594.26(42), 593.29(90), 576.18(15), 575.21(100), 533.21(21), 517.16(14), 515.35(14), 504.25(45), 503.23(251), 474.26(127), 473.24(1000), 455.29(23), 413.18(12), 383.28(131), 354.33(19), 353.26(238) | MS2[595.00@35]: 576.95(1000), 559.01(312), 541.08(130), 529.03(234), 523.08(105), 511.03(189), 499.15(105), 481.18(71), 475.12(166), 457.12(508), 409.16(37) | 593.1496 | **C_27_H_30_O_15_** | -2.7 |  |
| **27.64^a^** |  | MS[Full ms]596.02(64), 595.06(221), 451.41(53), 450.30(153), 447.27(57), 446.22(174), 430.30(126), 414.37(74), 374.33(111), 343.19(197), 342.21(1000), 297.23(91), 109.99(58) |  | MS2[342.20@35]: 343.10(63), 342.05(175), 313.07(22), 312.11(27), 311.06(169), 300.14(24), 299.04(158), 298.09(187), 297.05(1000), 285.15(19), 282.29(18), 279.20(43), 266.18(19), 265.16(150), 253.27(13) | 342.1699 | **C_20_H_23_O_4_N** | -0.2 |  |
| **28.98^a^** |  | MS[Full ms]432.18(16), 430.17(28), 424.38(41), 423.36(272), 422.34(1000), 390.49(68), 109.90(17) |  | MS2[422.30@35]: 423.19(318), 422.22(685), 391.32(149), 390.30(1000), 372.26(47), 358.16(20) | 422.2897 | **C_24_H_39_O_5_N** | -0.9 |  |
| **29.58^a^** |  | MS[Full ms]445.20(35), 444.20(141), 416.21(21), 347.32(31), 346.30(161), 345.40(212), 344.43(1000) |  | MS2[444.20@35]: 445.18(216), 444.16(374), 426.13(37), 385.17(151), 384.13(525), 371.16(156), 370.14(1000), 367.06(67), 366.15(299), 352.16(68), 338.30(73), 311.19(58), 310.18(344), 293.17(52), 292.14(255), 264.19(55) | 444.2369 | **C_25_H_33_O_6_N** | -2.6 |  |
| **29.58^a^** |  | MS[Full ms]445.20(35), 444.20(141), 416.21(21), 347.32(31), 346.30(161), 345.40(212), 344.43(1000) |  | MS2[344.40@35]: 345.28(256), 344.31(1000), 343.69(22), 326.39(60) | 344.2585 | **C_22_H_33_O_2_N** | 0.3 |  |
| **31.07** | MS[Full ms]1355.93(446), 1352.24(39), 1196.73(37), 1156.79(36), 1144.05(31), 1126.87(43), 1091.33(31), 1039.24(32), 999.24(35), 966.16(32), 810.19(35), 735.73(32), 631.33(37), 565.26(118), 564.27(270), 563.31(1000) | MS[Full ms]567.08(70), 566.10(260), 565.08(1000), 509.42(46), 508.41(135), 467.33(38), 466.37(108), 444.38(95), 430.32(84), 422.47(32), 351.01(43), 346.45(76), 345.53(158), 344.51(701), 343.69(56), 110.00(73) | MS2[563.30@35]: 564.26(88), 563.27(261), 546.15(35), 545.21(150), 503.25(82), 474.20(136), 473.20(609), 455.20(70), 444.24(182), 443.25(1000), 383.25(125), 353.23(206) |  | 563.1388 | **C_26_H_28_O_14_** | -3.2 |  |
| **31.39** | MS[Full ms]1382.45(314), 1375.04(60), 1239.58(33), 1127.91(62), 663.04(54), 565.29(39), 564.30(267), 563.26(1000) | MS[Full ms]567.14(81), 566.15(234), 565.11(1000), 488.30(96), 444.48(53), 430.45(106), 346.59(55), 345.56(118), 344.50(408), 328.45(169), 109.93(58) | MS2[563.30@35]: 564.19(85), 563.20(89), 546.22(85), 545.21(284), 504.28(174), 503.23(729), 474.29(243), 473.21(1000), 444.29(173), 443.23(816), 425.26(76), 413.28(55), 383.29(306), 353.31(363) |  | 563.1389 | **C_26_H_28_O_14_** | -3.1 |  |
| **32.27^a^** |  | MS[Full ms]565.07(25), 432.28(38), 431.30(253), 430.31(1000), 344.42(43) |  | MS2[430.30@35]: 431.18(355), 430.18(553), 413.17(104), 412.14(540), 395.13(199), 394.12(1000), 371.32(87), 370.26(361), 352.24(55), 335.14(37), 334.14(212), 310.19(36), 274.30(54), 190.87(30), 149.04(31) | 430.2222 | **C_24_H_31_O_6_N** | -0.5 |  |
| **32.68** | MS[Full ms]1214.83(61), 1127.21(65), 911.01(66), 905.07(82), 891.52(78), 565.31(116), 564.27(240), 563.26(1000) | MS[Full ms]566.09(59), 565.08(174), 432.43(49), 431.35(279), 430.36(1000), 429.68(45), 372.57(38), 345.57(31), 344.52(178), 122.31(37), 110.08(37) | MS2[563.30@35]: 564.20(131), 563.21(277), 545.19(155), 503.20(200), 485.29(54), 474.20(236), 473.19(1000), 455.18(203), 444.29(159), 443.24(746), 413.46(61), 383.28(274), 354.30(59), 353.26(294) |  | 563.1384 | **C_26_H_28_O_14_** | -4.0 |  |
| **35.24** | MS[Full ms]1485.56(336), 712.61(34), 711.41(66), 644.96(31), 579.31(60), 578.29(300), 577.33(1000), 433.24(40) | MS[Full ms]708.98(49), 581.05(68), 580.10(236), 579.09(1000), 534.38(61), 533.43(257), 532.39(818), 531.78(56), 435.01(63), 431.44(61), 430.43(110), 344.51(63), 273.20(82), 257.17(54), 109.94(49) | MS2[577.30@35]: 578.18(131), 577.23(475), 559.22(259), 503.26(250), 487.22(328), 473.18(322), 458.19(119), 457.24(1000), 413.28(67), 383.26(192), 353.34(129) | MS2[579.10@35]: 560.95(1000), 542.96(816), 525.05(338), 513.04(179), 495.03(101), 483.14(85), 481.11(42), 475.20(61), 459.03(143), 457.06(175), 441.08(146), 423.10(69) | 577.1540 | **C_27_H_30_O_14_** | -3.9 |  |
| **35.84^a^** |  | MS[Full ms]579.00(11), 460.30(47), 459.37(209), 458.38(1000) |  | MS2[458.40@35]: 459.19(226), 458.20(372), 399.19(113), 398.18(535), 381.11(74), 380.13(249), 371.15(219), 370.20(1000), 352.21(155), 311.17(63), 310.22(332), 293.15(56), 292.17(325), 264.20(65) | 458.2536 | **C_26_H_35_O_6_N** | -0.3 |  |
| **36.43^a^** |  | MS[Full ms]1128.17(94), 1101.94(107), 1100.83(206), 968.87(98), 845.77(85), 550.99(227), 465.40(345), 464.42(1000), 458.53(320), 420.28(103), 419.11(466), 387.59(152), 386.48(798), 258.31(202), 257.31(932) |  | MS2[464.42@35]: 465.23(120), 464.23(334), 433.24(111), 432.20(1000), 415.27(6), 414.34(39), 400.41(23), 354.11(10) | 464.3002 | **C_26_H_41_O_6_N** | -1.0 |  |
| **35.45** | MS[Full ms]1144.81(181), 1143.96(111), 1100.02(117), 1098.99(297), 662.84(105), 648.72(78), 617.13(98), 594.96(104), 586.17(67), 585.10(250), 578.43(118), 577.29(706), 550.25(254), 549.27(1000) |  | MS2[549.40@35]: 549.25(56), 430.20(103), 429.20(912), 418.16(42), 417.11(35), 387.25(34), 297.19(133), 256.27(132), 255.24(1000) |  | 549.1599 | **C_26_H_30_O_13_** | -2.7 |  |
| **35.51^a^** |  | MS[Full ms]580.18(93), 579.14(363), 533.39(63), 532.39(259), 447.06(188), 430.35(66), 420.11(176), 419.03(1000), 258.30(61), 257.22(440) |  | MS2[419.00@35]: 400.84(9), 352.75(11), 298.94(11), 297.21(14), 280.85(9), 257.04(1000) | 419.1327 | **C_21_H_22_O_9_** | -2.3 |  |
| **36.43^a^** | MS[Full ms]1481.12(136), 1386.33(142), 1369.82(162), 1320.13(144), 1299.43(145), 1288.78(1000), 1036.42(129), 719.36(148), 708.38(125), 707.26(486), 577.47(142), 563.05(135), 549.31(255), 417.23(301) | MS[Full ms]1128.17(94), 1101.94(107), 1100.83(206), 968.87(98), 845.77(85), 550.99(227), 465.40(345), 464.42(1000), 458.53(320), 420.28(103), 419.11(466), 387.59(152), 386.48(798), 258.31(202), 257.31(932) |  | MS2[419.00@35]: 297.18(13), 296.06(8), 257.04(1000), 256.35(11) | 419.1332 | **C_21_H_22_O_9_** | -1.1 |  |
| **36.59** | MS[Full ms]1375.39(78), 1374.65(139), 1101.23(175), 1100.23(437), 1099.19(1000), 594.95(100), 550.40(186), 549.42(621) |  | MS2[549.40@35]: 550.26(60), 549.24(155), 430.25(27), 429.23(149), 418.34(81), 417.22(336), 370.01(22), 357.14(38), 298.16(34), 297.16(234), 256.18(161), 255.17(1000) |  | 549.1600 | **C_26_H_30_O_13_** | -2.5 |  |
| **40.74^a^** |  | MS[Full ms]474.23(44), 473.27(234), 472.30(1000) |  | MS2[472.30@35]: 473.17(193), 472.21(225), 455.16(102), 454.15(462), 413.13(234), 412.17(1000), 394.26(247), 370.27(98), 352.32(204), 310.31(103) | 472.2332 | **C_26_H_33_O_7_N** | 0.5 |  |
| **44.41** | MS[Full ms]1396.43(49), 1217.33(37), 1198.01(36), 1183.23(38), 1075.23(35), 1009.36(235), 1008.40(450), 1007.34(1000), 976.50(34), 963.40(55), 832.95(36), 814.31(51), 813.34(138) |  | MS2[1007.30@35]: 1008.53(555), 1007.49(1000), 979.89(69), 963.57(270), 958.69(79), 946.66(155), 945.16(441), 900.38(72), 851.44(77), 844.63(94), 809.71(54), 783.57(223), 711.48(78), 705.21(52), 459.19(57) |  | 1007.5414 | **C_48_H_82_O_19_** | -1.8 |  |
| **45.53** | MS[Full ms]979.40(140), 978.43(413), 977.34(1000), 931.59(45) |  | MS2[931.60@35]: 909.33(72), 825.02(55), 799.31(1000), 769.47(260), 751.46(43), 704.46(42), 637.34(995), 520.14(49), 475.41(219) |  | 931.5259 | **C_47_H_80_O_18_** | -1.4 |  |
| **47.13** | MS[Full ms]1156.65(56), 1146.08(65), 1145.22(89), 1144.25(113), 1100.08(141), 1099.09(106), 662.84(116), 596.85(58), 595.95(278), 594.91(1000), 593.96(164), 585.09(112), 550.31(167), 549.29(657) | MS[Full ms]563.06(113), 553.03(60), 552.09(248), 551.06(1000), 419.21(120), 257.30(81) | MS2[549.30@35]: 550.21(70), 549.33(107), 430.17(21), 429.19(168), 418.23(51), 417.18(322), 400.37(11), 399.21(26), 357.27(24), 297.99(28), 297.14(252), 256.22(155), 255.22(1000), 254.26(12) | MS2[551.10@35]: 418.85(1000), 389.00(58), 257.06(624) | 549.1606 | **C_26_H_30_O_13_** | -1.4 |  |
| **48.02** | MS[Full ms]1392.62(32), 1318.73(45), 994.46(38), 993.40(139), 992.51(374), 991.40(1000), 945.54(45), 847.57(87), 846.54(255), 845.50(792) |  | MS2[945.50@35]: 799.29(577), 797.79(24), 783.40(1000), 782.69(23), 781.18(91), 765.43(219), 637.41(866), 619.45(274), 475.42(415) |  | 945.5404 | **C_48_H_82_O_18_** | -2.6 |  |
| **48.02** | MS[Full ms]1489.46(13), 1464.44(10), 1459.40(15), 1436.99(11), 1434.78(12), 1417.96(13), 1392.62(32), 1391.54(26), 1387.50(12), 1351.95(11), 1345.39(17), 1327.72(22), 1323.91(12), 1320.90(11), 1319.62(23), 1318.73(45), 1282.78(14), 1246.54(21), 1245.45(16), 1179.92(17), 1135.57(11), 1121.26(11), 1098.73(18), 1090.68(12), 1064.18(13), 1060.39(10), 996.34(11), 995.19(10), 994.46(38), 993.40(139), 992.51(374), 991.40(1000), 990.02(10), 945.54(45), 913.23(11), 848.46(13), 847.57(87), 846.54(255), 845.50(792), 799.69(10) |  | MS2[799.70@35]: 782.81(14), 738.12(12), 732.32(21), 679.00(22), 655.33(11), 652.48(17), 638.40(59), 637.30(1000), 619.35(35), 557.11(11), 507.27(11), 476.37(13), 475.55(217), 336.36(15) |  | 845.4895 | **C_42_H_72_O_14_** | -1.1 |  |
| **48.27** | MS[Full ms]1456.79(53), 1426.69(61), 1167.83(52), 1120.79(61), 1098.34(58), 993.58(90), 992.46(430), 991.43(1000), 990.27(54), 847.57(76), 846.48(262), 845.47(666), 792.49(58), 649.03(85), 595.00(55), 584.97(53), 550.28(62), 549.40(203) | MS[Full ms]794.25(57), 673.38(51), 672.46(93), 552.20(232), 551.18(1000), 550.19(55), 473.41(52), 472.38(110), 441.31(89), 423.44(171), 419.24(124), 257.31(106) | MS2[549.30@35]: 549.30(39), 430.16(162), 429.19(859), 417.15(111), 297.24(168), 256.23(153), 255.26(1000), 221.26(32), 219.24(48) | MS2[551.10@35]: 418.89(1000), 417.87(11), 389.07(59), 257.10(178) | 549.1606 | **C_26_H_30_O_13_** | -1.4 |  |
| **49.09^a^** | MS[Full ms]1461.78(32), 1415.95(32), 1412.02(42), 1389.01(32), 1370.86(33), 1321.36(39), 1276.14(52), 1247.11(34), 1242.79(42), 1237.40(33), 1222.12(31), 1204.18(35), 992.61(56), 973.81(31), 905.63(63), 904.71(106), 895.19(35), 894.14(38), 846.56(74), 845.62(52), 741.12(59), 477.19(51), 476.22(291), 475.16(1000), 474.54(53), 417.26(44) | MS[Full ms]433.23(26), 432.18(223), 431.12(1000), 420.32(30), 419.29(128), 270.39(24), 269.34(170) | MS2[475.20@35]: 428.69(47), 267.20(1000), 266.56(16) | MS2[431.10@35]: 432.27(1), 412.05(1), 402.78(1), 269.89(15), 269.09(1000), 267.73(1), 159.18(1) | 431.1344 | **C_22_H_22_O_9_** | 1.7 |  |
| **49.20^a^** | MS[Full ms]1444.63(104), 1025.00(104), 991.32(146), 979.81(111), 892.66(126), 848.00(240), 846.85(474), 834.95(177), 741.01(211), 697.33(151), 669.25(149), 476.17(195), 475.16(1000), 417.28(593) | MS[Full ms]554.98(45), 472.38(55), 433.21(39), 432.18(191), 431.10(1000), 421.27(39), 420.31(140), 419.27(723), 269.43(216), 257.28(83) |  | MS2[419.19@35]: 419.00(6), 257.02(1000), 256.39(17) | 419.1342 | **C_21_H_22_O_9_** | 1.3 |  |
| **50.98** | MS[Full ms]1426.87(22), 1390.88(21), 1118.94(31), 1118.25(22), 1065.54(25), 885.78(28), 794.99(39), 762.99(21), 725.34(47), 697.31(115), 696.37(260), 695.34(1000) | MS[Full ms]726.88(33), 698.10(49), 696.98(184), 502.48(54), 501.49(256), 500.47(1000), 257.35(36) | MS2[695.30@35]: 575.19(32), 550.25(137), 549.24(1000), 532.24(132), 531.26(515), 417.21(31), 399.28(43), 255.18(58) | MS2[697.00@35]: 534.68(109), 440.62(213), 439.98(60), 278.55(741), 277.19(44), 260.90(1000), 256.96(40), 243.02(64), 214.92(104), 196.96(41) | 695.1978 | **C_35_H_36_O_15_** | -0.5 |  |
| **51.14^a^** |  | MS[Full ms]727.89(40), 726.85(119), 697.97(32), 696.95(69), 502.31(56), 501.34(270), 500.34(1000) |  | MS2[500.34@35]: 501.18(140), 500.14(227), 483.10(125), 482.12(891), 441.20(192), 440.17(1000), 422.12(284), 413.25(121), 412.20(808), 398.27(128), 394.30(182), 380.20(186), 352.22(179), 334.22(83), 310.27(165), 292.17(85) | 500.2644 | **C_28_H_37_O_7_N** | 0.2 |  |
| **51.20** | MS[Full ms]1452.42(39), 1420.79(31), 826.54(39), 825.02(64), 792.98(51), 788.01(41), 760.97(32), 727.26(95), 726.30(342), 725.29(1000), 696.31(160), 695.30(593) | MS[Full ms]727.89(40), 726.85(119), 697.97(32), 696.95(69), 502.31(56), 501.34(270), 500.34(1000) | MS2[725.30@35]: 550.20(189), 549.20(1000), 532.26(172), 531.26(895), 417.19(42), 399.26(116), 255.30(133) | MS2[726.90@35]: 709.07(124), 596.03(63), 564.95(67), 564.11(51), 470.54(271), 338.64(45), 308.67(954), 307.67(36), 306.96(31), 290.90(1000), 290.02(49), 272.89(58), 257.05(33), 244.89(77), 212.02(33) | 725.2086 | **C_36_H_38_O_16_** | -0.2 |  |
| **55.15^a^** |  | MS[Full ms]1001.04(39), 848.31(32), 825.23(49), 337.65(31), 297.90(56), 258.23(152), 257.20(1000), 122.38(53), 110.07(55) |  | MS2[257.20@35]: 257.96(202), 257.00(905), 242.09(280), 240.04(122), 238.99(1000), 229.03(43), 215.03(38), 211.01(143), 163.09(111), 147.02(809), 137.08(644) | 257.0812 | **C_15_H_12_O_4_** | 1.4 |  |
| **55.94** | MS[Full ms]1315.16(79), 1000.56(287), 999.47(617), 938.36(83), 937.29(103), 904.79(69), 882.92(138), 859.46(64), 839.52(178), 838.55(319), 837.52(1000), 823.55(84), 694.38(62), 693.34(187), 692.32(777) | MS[Full ms]1003.34(158), 1002.29(410), 1001.26(1000), 987.33(61), 840.33(126), 839.28(296), 825.33(52), 695.24(59), 694.14(206), 663.47(73), 487.19(94), 470.62(98), 469.54(450), 460.03(79), 258.30(54), 257.25(95), 110.03(58) | MS2[999.50@35]: 880.43(17), 879.32(60), 838.43(204), 837.43(1000), 820.42(15), 819.54(170), 818.90(14), 661.59(50), 351.25(83) |  | 999.4437 | **C_48_H_72_O_22_** | -0.5 |  |
| **57.72** | MS[Full ms]1189.88(192), 1084.21(180), 1083.10(377), 1053.34(119), 999.20(101), 770.74(163), 726.22(347), 725.21(1000), 696.25(121), 695.27(490) | MS[Full ms]1002.16(350), 1001.10(656), 855.17(193), 728.27(502), 727.14(1000), 726.40(158), 515.41(208), 514.44(485), 494.69(216), 478.84(232), 454.53(190), 453.48(160), 376.22(154), 369.16(165), 341.28(654), 257.24(375), 122.35(204), 110.05(203) | MS2[725.20@35]: 550.17(25), 549.22(434), 532.29(36), 531.21(1000), 530.52(7), 399.36(27), 291.90(6), 255.20(41), 254.40(6) |  | 725.2088 | **C_36_H_38_O_16_** | 0.1 |  |
| **57.84** | MS[Full ms]1129.58(199), 1101.14(111), 1083.11(112), 1052.85(126), 825.13(101), 770.47(132), 726.34(275), 725.26(586), 696.37(271), 695.32(1000) | MS[Full ms]1225.09(276), 1132.27(192), 1131.22(222), 1001.65(211), 985.02(205), 855.24(321), 729.26(235), 728.07(447), 726.98(896), 698.41(306), 697.21(1000), 514.54(436), 500.52(200), 494.88(396), 493.98(193), 453.45(246), 341.16(361), 291.11(203), 257.33(558), 122.29(317), 109.86(282) | MS2[695.30@35]: 550.24(105), 549.21(638), 548.59(15), 532.24(101), 531.21(1000), 417.14(16), 399.26(45), 256.09(11), 255.33(51) | MS2[697.10@35]: 697.23(105), 696.23(263), 678.93(379), 616.44(172), 550.95(432), 534.94(1000), 534.24(123), 531.64(183), 516.84(382), 461.00(394), 419.01(123), 370.91(193), 352.86(184), 298.98(286), 260.95(170), 257.00(130) | 695.1978 | **C_35_H_36_O_15_** | -0.5 |  |
| **58.47** | MS[Full ms]1281.89(61), 1281.12(103), 1280.32(68), 953.56(122), 875.59(60), 855.60(130), 854.54(446), 853.53(1000) | MS[Full ms]909.13(30), 898.24(27), 897.18(82), 877.34(41), 874.53(27), 858.19(34), 857.25(87), 856.32(317), 855.25(1000), 839.48(21), 485.48(49), 469.41(28), 468.61(23), 467.75(27) | MS2[853.50@35]: 836.50(35), 835.34(62), 792.32(15), 791.46(61), 777.26(22), 678.42(63), 677.34(249), 659.39(46), 641.53(11), 541.62(11), 501.48(92), 457.51(16), 352.18(47), 351.12(1000), 333.26(23), 275.51(19) | MS2[855.30@35]: 878.83(786), 865.00(286), 857.05(268), 854.61(225), 840.11(471), 827.51(339), 826.81(196), 816.18(265), 796.89(154), 733.11(98), 639.01(502), 632.85(1000), 297.13(347) | 853.3874 | **C_42_H_62_O_18_** | 1.2 |  |
| **59.16^b^** | MS[Full ms]1373.81(36), 1337.91(42), 1114.46(31), 1113.40(90), 1083.77(32), 1063.63(44), 1052.78(36), 1051.60(67), 1032.69(50), 1031.58(149), 1030.67(639), 1029.58(973), 1013.70(49), 939.50(32), 644.38(93), 516.71(105), 515.76(292), 514.71(1000), 464.66(37) |  | MS2[514.70@40]: 516.55(51), 515.56(343), 514.57(1000), 450.54(13), 449.42(6), 448.56(9), 433.74(8), 432.69(16), 431.65(2), 430.75(13), 426.44(5), 412.41(10), 406.61(5), 402.56(6), 386.42(8), 384.48(5), 370.85(4) |  | 514.2837 | **C_26_H_45_O_7_NS** | -1.4 |  |
| **59.39** | MS[Full ms]1487.26(26), 1477.39(40), 1476.57(65), 1475.66(92), 1083.32(65), 1010.35(36), 1005.47(64), 995.88(27), 986.39(55), 985.50(143), 984.56(461), 983.63(1000), 983.02(47) | MS[Full ms]1039.22(21), 1007.41(27), 1005.25(27), 988.41(27), 987.49(127), 986.49(378), 985.46(1000) | MS2[983.60@35]: 863.24(24), 845.36(13), 822.35(179), 821.45(1000), 820.81(11), 804.48(35), 803.49(167), 645.56(37), 351.23(72) | MS2[985.50@35]: 999.96(57), 970.52(73), 967.19(111), 941.31(141), 937.86(35), 909.29(73), 824.38(42), 822.80(34), 812.52(51), 809.07(609), 711.80(36), 664.29(64), 647.17(70), 633.21(145), 615.16(1000), 549.53(37), 471.01(300), 453.12(668), 434.17(91), 407.28(72) | 983.4504 | **C_48_H_72_O_21_** | 1.1 |  |
| **60.88** | MS[Full ms]913.39(33), 848.26(31), 847.30(136), 846.33(395), 845.24(1000), 837.65(33), 799.62(25) |  | MS2[799.60@35]: 642.03(17), 638.34(264), 637.34(919), 619.37(43), 566.12(21), 549.46(53), 476.48(221), 475.51(1000), 459.70(11) |  | 799.4850 | **C_42_H_72_O_14_** | 0.1 |  |
| **62.11** | MS[Full ms]1253.04(48), 1156.24(73), 1155.42(255), 1154.45(735), 1153.78(563), 1153.17(1000), 1149.00(49), 1130.54(78), 1107.64(63), 1046.86(44), 994.17(46) |  | MS2[1107.60@35]: 946.45(817), 945.59(1000), 784.55(225), 783.56(493), 766.48(29), 765.40(45), 621.53(92), 459.55(54), 322.98(21) |  | 1107.5949 | **C_54_H_92_O_23_** | -0.7 |  |
| **62.16^b^** | MS[Full ms]1380.45(21), 1378.65(54), 1083.56(23), 1082.63(30), 1081.72(60), 1020.77(27), 1019.64(37), 1000.52(63), 999.60(196), 998.68(440), 997.64(1000), 996.71(26), 500.76(43), 499.68(154), 498.69(510) |  | MS2[498.70@40]: 500.68(20), 499.59(364), 498.57(1000), 434.44(12), 433.52(2), 432.42(3), 414.70(3), 398.19(2), 373.59(5), 372.77(5), 355.50(2) |  | 498.2895 | **C_26_H_45_O_6_NS** | -0.6 |  |
| **62.41^a^** |  | MS[Full ms]1326.37(59), 1325.26(62), 861.50(93), 858.99(64), 840.66(141), 839.77(127), 838.91(112), 664.35(63), 663.40(86), 487.50(69), 471.44(165), 470.51(398), 469.51(1000) |  | MS2[469.70@35]: 452.20(182), 451.22(1000), 434.14(129), 433.14(570), 423.08(133), 405.12(262), 260.96(112), 237.05(259), 233.01(196), 219.00(92), 217.09(139), 190.96(74), 187.07(83), 175.09(600), 173.04(215) | 469.3314 | **C_30_H_44_O_4_** | 0.3 |  |
| **62.46** | MS[Full ms]1392.43(47), 1391.65(77), 1268.32(37), 1267.62(61), 1258.67(78), 1257.72(291), 1256.74(430), 1234.38(34), 1153.17(40), 972.69(49), 839.55(153), 838.60(377), 837.53(1000) | MS[Full ms]1325.88(32), 1325.17(40), 1307.72(39), 867.01(48), 861.48(71), 858.30(39), 840.54(38), 839.88(49), 839.15(91), 664.68(33), 663.52(118), 487.51(98), 471.89(38), 470.85(294), 469.66(1000) | MS2[837.50@35]: 820.25(40), 819.39(125), 775.48(108), 661.43(181), 643.54(37), 485.39(79), 352.19(51), 351.13(1000) |  | 837.3923 | **C_42_H_62_O_17_** | 1.0 |  |
| **63.27** | MS[Full ms]1256.19(42), 1255.04(156), 1191.38(57), 1189.48(45), 1126.37(37), 1125.39(149), 1124.38(529), 1123.42(1000), 1122.37(46), 1100.71(33), 1078.73(33), 1077.82(32) |  | MS2[1078.70@35]: 946.48(1000), 945.54(893), 916.39(118), 915.45(97), 784.53(331), 783.54(589), 766.56(64), 765.47(156), 622.48(49), 621.38(113), 459.51(53) |  | 1077.5851 | **C_53_H_90_O_22_** | 0.0 |  |
| **63.63** | MS[Full ms]1457.79(69), 1448.10(58), 1413.63(86), 1404.53(74), 1380.11(57), 1330.12(54), 1256.69(110), 1219.14(54), 1193.63(127), 1125.28(91), 1124.34(175), 1123.39(243), 1073.48(119), 1072.49(107), 1028.29(56), 1027.10(51), 861.82(52), 859.43(96), 839.63(202), 838.60(352), 837.56(1000), 823.65(58) | MS[Full ms]1031.13(68), 1030.16(126), 1029.11(313), 985.33(67), 941.53(62), 883.29(66), 861.45(119), 858.21(60), 857.22(51), 856.41(88), 855.60(74), 842.38(86), 841.20(193), 840.51(91), 839.90(99), 839.22(218), 663.37(62), 616.57(55), 487.48(54), 480.35(127), 470.59(328), 469.47(1000), 452.43(66), 451.64(54), 435.02(71), 271.13(79) | MS2[837.50@35]: 820.50(31), 819.46(187), 775.47(251), 661.34(123), 643.50(70), 581.38(22), 486.40(22), 485.46(156), 355.38(20), 352.29(13), 351.19(1000), 332.85(15), 307.17(27), 259.24(17) |  | 837.3912 | **C_42_H_62_O_17_** | -0.3 |  |
| **64.37** | MS[Full ms]1498.52(86), 1497.34(47), 1496.60(77), 1474.44(22), 1471.54(31), 1433.86(21), 1433.24(40), 1429.91(21), 1379.98(32), 1336.45(28), 1316.28(23), 1271.15(27), 1258.67(22), 1229.00(25), 1222.37(31), 1195.22(41), 1192.55(58), 1191.29(31), 1186.06(32), 1171.43(24), 1170.56(26), 1166.23(25), 1159.70(22), 1126.26(22), 1125.35(238), 1124.37(578), 1123.40(1000), 1122.62(80), 1113.51(41), 1109.00(22), 1100.58(31), 1077.59(21), 1069.45(33), 1061.82(23), 1058.64(26), 1040.60(23), 1039.81(93), 1032.08(32), 1000.52(54), 958.55(21), 957.65(70), 955.82(61), 955.09(42), 939.54(59), 846.96(32), 839.50(25), 838.53(34), 837.51(88), 829.49(27) |  | MS2[1078.70@35]: 947.28(40), 946.53(744), 945.60(1000), 944.94(56), 916.53(162), 915.53(124), 784.58(320), 783.59(607), 766.51(60), 765.53(92), 621.35(100), 459.33(23) |  | 1077.5846 | **C_53_H_90_O_22_** | -0.5 | |
| **64.41^b^** | MS[Full ms]1449.46(12), 1448.59(23), 1434.80(23), 1433.93(59), 1432.86(92), 1014.60(12), 1013.28(13), 952.78(17), 951.66(44), 932.47(26), 931.55(144), 930.55(434), 929.53(1000), 465.57(65), 464.60(268) |  | MS2[464.60@35]: 465.48(436), 464.46(1000), 447.49(46), 446.53(37), 421.57(99), 420.59(623), 419.32(15), 418.49(32), 403.54(56), 402.26(73), 389.38(260), 385.18(26), 384.44(175) |  | 464.3018 | **C_26_H_43_O_6_N** | 0.3 |  |
| **64.51** | MS[Full ms]1495.49(216), 1494.63(103), 1435.34(220), 1434.67(301), 1433.95(501), 1374.99(128), 1124.46(283), 1123.39(415), 1074.80(86), 1040.09(123), 1039.26(255), 1001.80(172), 1000.93(293), 1000.24(120), 956.80(407), 955.79(1000) |  | MS2[955.80@35]: 835.38(27), 794.49(390), 793.52(1000), 775.54(36), 731.64(34), 613.63(22), 595.52(16), 569.66(32), 523.56(38) |  | 955.4903 | **C_48_H_76_O_19_** | -0.5 |  |
| **66.18** | MS[Full ms]1286.63(47), 1283.62(55), 1268.79(55), 1267.72(87), 1259.08(41), 1258.31(157), 1257.48(336), 1256.69(518), 937.07(76), 884.25(44), 883.35(111), 882.63(114), 840.52(53), 839.56(95), 838.55(437), 837.58(1000) | MS[Full ms]862.57(11), 861.46(27), 859.34(12), 858.59(15), 842.68(11), 841.58(121), 840.50(317), 839.47(1000), 487.76(17), 470.65(18), 469.64(39), 460.53(12) | MS2[837.60@35]: 820.43(23), 819.38(42), 793.27(12), 775.50(62), 662.36(28), 661.46(122), 625.08(14), 485.46(16), 352.24(55), 351.13(1000), 289.29(13) |  | 837.3921 | **C_42_H_62_O_17_** | 0.8 |  |
| **66.92** | MS[Full ms]1400.02(59), 1398.89(77), 1166.51(56), 1067.17(91), 991.00(68), 989.62(81), 970.48(60), 969.58(115), 968.61(455), 967.61(1000), 839.27(68), 837.48(124) |  | MS2[967.60@35]: 994.37(6), 968.16(6), 950.55(9), 949.57(51), 905.22(10), 833.45(14), 645.30(29), 498.24(108), 497.17(1000), 479.35(8), 435.17(50), 409.28(9), 407.14(25), 381.23(12), 340.19(23), 339.17(163), 322.27(8), 321.09(61) |  | 967.4548 | **C_48_H_72_O_20_** | 0.4 |  |
| **67.57** | MS[Full ms]1489.95(53), 1479.80(67), 1424.22(61), 1084.93(113), 1032.55(119), 1031.61(201), 1030.68(705), 1029.90(151), 1029.25(99), 1028.42(119), 1022.93(70), 1021.91(66), 1008.51(70), 1007.52(89), 988.47(79), 987.69(63), 986.58(380), 985.54(1000), 837.43(62) | MS[Full ms]1009.41(60), 989.35(121), 988.31(410), 987.26(1000), 842.44(58), 841.45(289) | MS2[985.50@35]: 968.40(28), 967.46(138), 923.51(43), 822.33(8), 821.36(6), 809.28(9), 664.24(8), 663.33(51), 498.18(106), 497.17(1000), 487.68(7), 453.01(9), 451.25(10), 435.43(8), 407.15(10), 404.73(6), 380.79(7), 340.28(8), 339.10(105), 321.14(25) |  | 985.4656 | **C_48_H_74_O_21_** | 0.6 |  |
| **67.71** | MS[Full ms]1086.75(73), 1031.35(96), 1030.38(135), 985.55(190), 839.45(146), 838.47(418), 837.47(1000), 795.60(86), 794.52(87), 793.47(345) | MS[Full ms]987.22(72), 861.37(50), 841.25(159), 840.28(360), 839.27(1000), 663.48(87), 470.44(44), 469.44(122) | MS2[837.60@35]: 859.76(11), 834.14(16), 820.36(31), 819.49(104), 793.32(51), 776.38(16), 775.46(88), 686.70(13), 661.54(52), 643.28(23), 626.42(11), 352.19(36), 351.13(1000), 261.14(12) |  | 837.3914 | **C_42_H_62_O_17_** | 0.0 |  |
| **68.19^b^** | MS[Full ms]1400.68(14), 1386.67(13), 1385.94(26), 1384.91(37), 919.72(12), 900.45(38), 899.53(152), 898.51(485), 897.50(1000), 449.61(33), 448.59(163) |  | MS2[448.60@35]: 449.41(308), 448.42(1000), 447.45(32), 405.44(32), 404.45(407), 386.48(144) |  | 448.3065 | **C_26_H_43_O_5_N** | -0.8 |  |
| **68.61** | MS[Full ms]1294.68(38), 1284.45(33), 1276.82(34), 1258.81(37), 1257.82(104), 1256.94(150), 1235.10(35), 1221.58(67), 937.17(58), 882.75(75), 881.88(48), 860.40(36), 859.50(116), 839.51(158), 838.54(392), 837.58(1000) | MS[Full ms]1225.23(17), 1224.21(39), 1223.16(51), 893.11(16), 862.42(23), 861.37(48), 842.36(26), 841.33(96), 840.35(340), 839.34(1000), 470.60(23), 469.62(58), 460.08(22) | MS2[837.60@35]: 820.27(18), 819.36(113), 801.27(8), 777.42(7), 776.45(24), 775.46(64), 749.10(7), 662.33(27), 661.50(124), 643.39(24), 599.52(16), 555.48(11), 553.76(7), 486.33(8), 485.31(14), 352.27(36), 351.11(1000), 332.95(6), 289.51(7), 244.53(9) |  | 837.3914 | **C_42_H_62_O_17_** | 0.9 |  |
| **69.06** | MS[Full ms]1280.02(16), 1260.09(16), 1244.86(17), 1233.97(25), 995.09(12), 920.26(11), 919.58(17), 888.92(11), 854.53(12), 823.52(149), 822.57(518), 821.57(1000), 795.54(16) | MS[Full ms]1470.07(44), 1469.24(63), 1295.94(97), 1295.12(125), 1294.43(203), 1293.52(249), 1277.52(71), 1276.82(53), 1276.19(106), 1275.53(88), 846.58(61), 845.47(94), 826.64(49), 825.45(196), 824.49(258), 823.50(618), 649.71(47), 648.71(91), 647.60(243), 473.47(56), 472.41(223), 471.44(318), 456.49(43), 455.68(104), 454.75(471), 453.68(1000) | MS2[821.50@35]: 803.35(120), 778.38(21), 759.53(48), 688.35(14), 645.49(83), 627.54(36), 352.25(18), 351.12(1000), 332.78(28), 288.88(28) |  | 821.3978 | **C_42_H_62_O_16_** | 1.6 |  |
| **69.11^a^** |  | MS[Full ms]1295.80(96), 1294.85(207), 1293.78(341), 1276.64(111), 1275.83(80), 845.69(115), 825.73(111), 824.77(137), 823.79(423), 823.03(82), 648.61(103), 647.60(157), 472.72(134), 471.63(368), 455.62(104), 454.83(219), 453.76(1000) |  | MS2[471.50@35]: 453.19(1000), 435.25(145), 426.16(120), 425.21(477), 408.26(87), 407.15(343), 389.15(109), 317.10(265), 291.05(84), 262.97(344), 235.02(381), 216.99(190), 199.06(93), 191.10(89), 189.08(414), 175.00(112), 161.03(94) | 471.3468 | **C_30_H_46_O_4_** | -0.2 |  |
| **70.53^b^** | MS[Full ms]1496.43(54), 1482.67(22), 1218.63(22), 1099.10(24), 1082.57(24), 1081.60(68), 1020.69(39), 1019.57(46), 1000.52(45), 999.54(205), 998.60(478), 997.56(1000), 628.42(52), 566.45(30), 500.69(65), 499.73(191), 498.67(606) |  | MS2[498.70@40]: 500.51(6), 499.63(284), 498.63(1000), 480.88(2), 435.67(2), 434.69(6), 432.87(6), 429.88(2), 376.69(2) |  | 498.2895 | **C_26_H_45_O_6_NS** | 0.0 |  |
| **70.55** | MS[Full ms]1354.33(51), 1339.81(64), 1269.50(53), 1164.54(53), 1067.05(60), 990.51(58), 989.52(150), 970.53(58), 969.59(120), 968.54(376), 967.57(1000), 921.30(73), 843.69(78), 823.58(141), 822.57(349), 821.57(942) |  | MS2[967.60@35]: 950.50(52), 949.44(84), 924.37(7), 906.67(18), 905.29(20), 799.47(6), 646.50(15), 645.51(19), 628.41(24), 583.11(8), 540.36(10), 539.42(8), 498.19(139), 497.18(1000), 496.50(8), 479.12(7), 453.16(6), 435.30(28), 423.34(9), 407.24(16), 344.89(10), 339.15(168), 321.16(41) |  | 967.4557 | **C_48_H_72_O_20_** | 1.3 |  |
| **70.88** | MS[Full ms]1015.82(107), 1014.70(216), 991.57(105), 971.60(166), 970.62(461), 969.56(1000), 838.55(112), 837.48(224), 824.59(124), 823.64(268), 822.53(199), 821.53(575) | MS[Full ms]994.45(164), 989.07(173), 988.23(370), 972.21(387), 971.13(677), 842.24(214), 840.42(632), 839.31(826), 826.45(180), 825.31(341), 824.21(348), 823.29(596), 473.42(164), 471.48(371), 456.57(208), 455.48(823), 454.57(324), 453.53(1000), 438.57(164), 437.54(361) | MS2[969.60@35]: 952.45(61), 951.46(81), 907.44(26), 647.42(12), 629.36(13), 498.24(156), 497.17(1000), 479.12(29), 435.36(44), 407.28(16), 340.03(13), 339.14(121), 321.15(41) |  | 969.4712 | **C_48_H_74_O_20_** | 1.2 |  |
| **71.25** | MS[Full ms]1099.86(35), 1075.17(58), 991.85(31), 921.48(39), 907.36(121), 838.45(35), 837.65(36), 830.68(31), 829.46(63), 823.65(36), 822.68(64), 821.55(228), 809.56(70), 808.60(427), 807.64(1000) | MS[Full ms]840.24(118), 839.14(195), 831.38(192), 823.14(173), 811.27(169), 810.34(394), 809.34(1000), 633.32(165), 471.37(134), 457.39(209), 454.47(116), 453.50(307), 445.01(137), 440.56(302), 439.52(988), 269.30(757) | MS2[807.60@35]: 789.48(48), 746.53(13), 745.50(30), 631.48(39), 352.21(47), 351.15(1000), 333.01(25) | MS2[809.30@35]: 810.88(723), 809.75(569), 633.24(601), 629.53(692), 313.01(1000) | 807.4180 | **C_42_H_64_O_15_** | 0.9 |  |
| **71.75^a^** |  | MS[Full ms]1294.40(36), 1293.49(60), 1275.58(33), 1262.45(33), 845.31(50), 843.44(34), 842.34(35), 824.14(61), 823.27(81), 648.54(39), 647.60(80), 472.68(40), 471.96(52), 471.34(77), 455.78(68), 454.73(264), 453.67(1000) |  | MS2[453.70@35]: 436.18(220), 435.15(1000), 407.16(574), 389.22(243), 299.11(81), 285.04(85), 245.09(88), 241.20(93), 235.05(428), 217.07(193), 189.10(211), 175.08(119) | 453.3365 | **C_30_H_44_O_3_** | 0.4 |  |
| **71.80** | MS[Full ms]1484.36(22), 1483.42(24), 1259.53(37), 1234.04(34), 1233.43(66), 1232.58(108), 921.06(26), 823.52(85), 822.59(330), 821.53(1000) | MS[Full ms]1294.40(36), 1293.49(60), 1275.58(33), 1262.45(33), 845.31(50), 843.44(34), 842.34(35), 824.14(61), 823.27(81), 648.54(39), 647.60(80), 472.68(40), 471.96(52), 471.34(77), 455.78(68), 454.73(264), 453.67(1000) | MS2[821.50@35]: 803.35(93), 777.43(39), 759.33(60), 687.10(16), 645.39(76), 627.16(50), 470.34(13), 352.18(19), 351.12(1000), 332.88(23) |  | 821.3979 | **C_42_H_62_O_16_** | 1.7 |  |
| **71.85^a^** |  | MS[Full ms]1275.20(52), 845.41(77), 823.18(60), 647.28(98), 471.45(149), 454.41(297), 453.40(1000) |  | MS2[471.50@35]: 453.22(330), 435.15(216), 425.13(239), 407.12(200), 317.13(85), 262.91(86), 245.03(126), 234.97(1000), 217.03(325), 191.06(106), 189.10(368), 175.03(258) | 471.3466 | **C_30_H_46_O_4_** | -0.6 |  |
| **72.66** | MS[Full ms]1353.47(33), 1244.53(65), 1233.90(71), 1232.63(55), 921.29(43), 909.62(32), 843.52(109), 823.47(103), 822.54(511), 821.55(1000) | MS[Full ms]917.91(56), 850.18(58), 846.44(55), 845.36(151), 843.21(66), 842.34(81), 841.23(54), 840.21(181), 824.32(101), 823.27(373), 472.55(110), 471.53(157), 455.41(57), 454.47(269), 453.41(1000) | MS2[821.50@35]: 804.42(37), 803.50(135), 802.88(23), 760.35(28), 759.40(113), 646.44(25), 645.35(38), 627.35(93), 351.97(51), 351.13(1000), 263.30(11) |  | 821.3977 | **C_42_H_62_O_16_** | 1.4 |  |
| **72.99^b^** | MS[Full ms]1329.54(36), 1263.00(151), 1262.05(107), 899.65(89), 840.14(33), 838.65(68), 837.82(207), 821.69(50), 817.72(138), 816.65(458), 815.61(1000), 454.33(79), 453.41(357), 425.07(44), 407.53(60) |  | MS2[407.50@40]: 425.85(165), 424.83(1000), 423.97(344), 423.04(171), 421.96(127), 420.08(82), 408.58(106), 407.60(558), 406.67(399), 405.28(184), 403.20(59), 390.51(121), 389.55(410), 387.62(54), 371.30(34), 342.54(33) |  | 407.2803 | **C_24_H_40_O_5_** | 0.7 |  |
| **73.11** | MS[Full ms]1343.52(41), 1279.56(50), 1123.70(49), 989.19(43), 921.46(105), 868.07(78), 866.90(148), 865.84(118), 843.54(85), 824.57(48), 823.59(61), 822.56(312), 821.46(1000) | MS[Full ms]846.33(66), 845.28(115), 825.25(141), 824.28(409), 823.23(1000), 499.43(76), 494.31(125), 472.48(66), 471.52(117), 454.49(156), 453.45(378), 417.03(70), 318.40(89), 297.03(65) | MS2[821.50@35]: 804.35(12), 803.31(109), 759.57(12), 645.45(103), 627.94(19), 352.10(25), 351.10(1000) |  | 821.3979 | **C_42_H_62_O_16_** | 1.7 |  |
| **74.11** | MS[Full ms]1480.95(71), 1245.24(74), 1236.41(181), 1235.64(112), 1223.46(85), 1213.43(167), 1212.22(130), 953.66(103), 868.80(323), 867.52(91), 845.58(125), 824.48(454), 823.48(1000), 822.63(395), 778.84(80), 777.54(252) | MS[Full ms]847.51(64), 827.38(116), 826.39(328), 825.39(1000), 648.61(48), 647.64(92), 472.77(32), 471.71(61), 454.09(60), 453.28(74) | MS2[823.50@35]: 806.34(17), 805.50(85), 779.32(17), 761.41(33), 648.32(14), 647.39(101), 352.17(55), 351.12(1000), 333.06(20), 289.13(12) |  | 823.4128 | **C_42_H_64_O_16_** | 0.8 |  |
| **74.11** | MS[Full ms]1236.41(181), 1235.64(112), 1223.46(85), 1213.43(167), 1212.22(130), 953.66(103), 868.80(323), 867.52(91), 845.58(125), 824.48(454), 823.48(1000), 822.63(395), 777.54(252) |  | MS2[777.60@35]: 778.45(97), 716.51(60), 715.52(213), 628.51(317), 627.49(1000), 537.58(214), 469.53(41) |  | 777.4070 | **C_41_H_62_O_14_** | 0.4 |  |
| **75.07^a^** |  | MS[Full ms]1426.26(84), 1425.33(60), 823.12(64), 801.34(87), 798.66(77), 796.25(69), 770.53(71), 649.43(53), 648.50(280), 647.41(1000), 472.57(69), 471.36(198), 454.56(82), 453.53(283) |  | MS2[647.60@35]: 471.30(12), 454.23(17), 453.19(1000), 452.34(9), 435.24(15), 407.35(7) | 647.3789 | **C_36_H_54_O_10_** | -0.1 |  |
| **75.00** | MS[Full ms]923.04(78), 845.32(134), 825.06(115), 824.07(335), 823.00(1000), 821.78(197), 813.46(81), 778.58(85), 777.62(335) |  | MS2[777.60@35]: 778.33(249), 777.52(109), 716.46(89), 715.45(348), 645.40(78), 628.50(143), 627.47(455), 610.25(91), 609.32(190), 601.56(80), 583.52(190), 538.50(378), 537.48(1000), 469.48(78) |  | 777.4077 | **C_41_H_62_O_14_** | 1.3 |  |
| **75.89** | MS[Full ms]1359.03(54), 1152.99(65), 1089.34(62), 907.24(65), 905.18(74), 895.18(68), 827.41(102), 823.51(69), 822.56(110), 821.54(95), 807.47(122), 806.53(405), 805.53(1000) |  | MS2[805.50@35]: 788.25(6), 787.43(125), 786.57(8), 743.38(28), 671.38(13), 669.23(8), 629.59(20), 628.59(7), 351.15(1000), 333.42(13), 288.94(20) |  | 805.4033 | **C_42_H_62_O_15_** | 2.1 |  |
| **76.89^b^** | MS[Full ms]1386.81(24), 1385.90(37), 1384.93(31), 919.66(22), 900.52(34), 899.54(132), 898.57(378), 897.57(1000), 449.60(22), 448.62(116) |  | MS2[448.60@35]: 449.51(816), 448.47(1000), 430.51(79), 404.54(190), 387.72(85), 386.54(402) |  | 448.3068 | **C_26_H_43_O_5_N** | -1.0 |  |
| **76.97** | MS[Full ms]1344.10(64), 1265.32(53), 1211.71(119), 907.22(86), 829.61(81), 809.45(140), 808.54(371), 807.60(1000), 806.76(52), 805.55(109) |  | MS2[807.60@35]: 789.39(53), 745.47(46), 631.52(31), 352.18(44), 351.16(1000), 333.24(20) |  | 807.4186 | **C_42_H_64_O_15_** | 1.7 |  |
| **78.68^a^** |  | MS[Full ms]823.05(71), 471.33(61), 469.47(258), 462.06(176), 461.32(236), 363.71(61), 342.90(111), 342.28(71), 334.54(140), 295.16(221), 278.27(179), 277.19(1000), 177.29(154), 137.15(69) |  | MS2[277.18@35]: 259.07(26), 176.97(1000), 141.06(13), 137.04(13) | 277.1804 | **C_17_H_24_O_3_** | 2.1 |  |

**^a^** The retention time in positive base peak mass spectrum.

**^b^** The retention time and detailed MS data of ZSIN.

Table S2. Detailed HRMS^2^ data of FSIN or ZSIN for metabolites identification.

| **Rt (min)** | **MS^2^ Pos/Neg** | **MW (HR-MS)** | **Formula** | **Intensity** | **Intensity of control** |
| --- | --- | --- | --- | --- | --- |
| 14.26^b^ | MS2[394.3@35]: 394.2600(1000), 376.2492(299) | 394.2591 | C_22_H_35_O_5_N | 98022 | 6491 |
| 15.11^b^ | MS2[326.2@35]: 326.1738(1000) | 326.1751 | C_20_H_23_O_3_N | 4988 | 875 |
| 18.15^b^ | MS2[406.2@35]: 406.2220(1000), 370.2010(67) | 406.2225 | C_22_H_31_O_6_N | 28827 | 3730 |
| 19.75^b^ | MS2[436.3@35]: 336.2688(1000), 418.2583(361) | 436.2698 | C_24_H_37_O_6_N | 104116 | 12002 |
| 28.94^b^ | MS2[488.2@35]: 488.2286(1000), 428.2076(147), 350.1755(144), 489.2304(99), 308.1653(86), 368.1859(72), 470.2157(62), 290.1545(56) | 488.2281 | C_26_H_33_O_8_N | 53574 | 2103 |
| 29.29^b^ | MS2[504.2@35]: 504.2233(1000), 444.2014(140), 505.2271(98), 486.2109(84), 324.1594(56) | 504.2227 | C_26_H_33_O_9_N | 8683 | 1695 |
| 29.57^b^ | MS2[448.3@35]: 448.2706(1000) | 448.2697 | C_25_H_37_O_6_N | 112493 | 8356 |
| 33.30^b^ | MS2[434.3@35]: 434.2903(1000), 402.2637(355) | 434.2898 | C_25_H_39_O_5_N | 15532 | 3051 |
| 39.64^b^ | MS2[461.1@35]: 461.3008(73), 285.0761(1000), 270.0518(77) | 461.1081 | C_22_H_20_O_11_ | 25202 | 2659 |
| 40.63^b^ | MS2[516.3@35]: 517.2618(138), 516.2590(1000), 428.2059(64), 350.1754(54) | 516.2594 | C_28_H_37_O_8_N | 75170 | 4483 |
| 41.18 | MS2[533.2@-92]: 140.1159(203), 139.1137(1000), 135.0459(203), 121.0292(406) | 533.1691 | C_26_H_30_O_12_ | 117492 | 3388 |
| 42.06^b^ | MS2[456.2@35]:456.2382(1000), 396.2168(384) | 456.2382 | C_26_H_33_O_6_N | 13495 | 1537 |
| 42.20^b^ | MS2[273.1@35]: 273.0751(83), 214.9200(55), 153.0180(1000), 147.0455(136), 119.0486(136), 68.9985(55), 67.0206(55) | 273.0760 | C_15_H_12_O_5_ | 17013 | 3419 |
| 42.20^b^ | MS2[449.1@35]: 273.0763(1000);), 153.0181(338);), 147.0438(109) | 449.1079 | C_21_H_20_O_11_ | 31123 | 709 |
| 42.55^b^ | MS2[463.1@35]: 287.0926(1000), 269.0815(50), 245.0811(384), 193.0496(198), 139.0387(54), 121.0289(214), 107.0494(58) | 463.1237 | C_22_H_22_O_11_ | 102617 | 1236 |
| 42.56^b^ | MS2[287.1@35]: 245.0792(252), 204.8872(252), 181.0659(252), 178.0270(252), 139.0415(379), 121.0285(1000) | 287.0916 | C_16_H_14_O_5_ | 19059 | 687 |
| 43.27^b^ | MS2[486.2@35]: 486.2492(1000), 468.2379(81), 426.2281(74), 412.2118(69), 408.2176(57), 310.1803), (87) | 486.2490 | C_27_H_35_O_7_N | 172464 | 15123 |
| 47.13^b^ | MS2[445.1@35]: 269.0816(1000) | 445.1131 | C_22_H_20_O_10_ | 126437 | 9170 |
| 49.07^b^ | MS2[433.1@35]: 257.0822(1000), 239.0711(64), 147.0447(160), 137.0239(242) | 433.1132 | C_21_H_20_O_10_ | 229824 | 2842 |
| 50.71^b^ | MS2[255.1@35]: 255.0649(1000), 237.0528(144), 227.0710(144), 199.0744(356), 181.0646(283), 153.0694(217), 152.0617(283), 137.0235(283), 91.0555(144) | 255.0653 | C_15_H_10_O_4_ | 6314 | 768 |
| 54.90^b^ | MS2[755.2@35]: 756.2481(288), 755.2444(662), 674.2744(366), 673.2713(571), 592.3007(80), 591.2988(240), 398.1020(193), 397.0997(1000), 316.1304(160), 315.1273(871) | 755.2426 | C_34_H_42_O_19_ | 15549 | 466 |
| 55.67^b^ | MS2[580.2@35]: 580.2907(67), 580.2573(138), 369.1739(371), 288.2063(140), 287.2013(1000), 228.0333(174), 137.0599(243) | 580.2416 | C_28_H_37_O_12_N | 18745 | 2485 |
| 55.95 | MS2[445.1@-92]: 254.0572(390), 253.0512(446), 239.0366(221), 225.0554(667), 224.0468(169), 212.0426(221), 211.0402(1000), 210.0326(390), 209.0608(779), 208.0538(221), 198.0690(169), 197.0610(221), 195.0471(221), 185.0624(390), 184.0529(277), 183.0451(502), 182.0375(446), 181.0672(277), 180.0566(169), 171.0481(221), 167.0503(277), 165.0720(221), 156.0566(169), 155.0505(169), 133.0291(169), 121.0322(169) | 445.1137 | C_22_H_22_O_10_ | 184121 | 8231 |
| 56.00^b^ | MS2[271.1@35]: 137.0598(1000), 135.0437(158), 123.0444(117), 147.0435(117), 161.0598(117), 109.0655(90), 79.0560(51) | 271.0967 | C_16_H_14_O_4_ | 37070 | 3629 |
| 65.44^a,b^ | MS2[432.3@35]: 414.3017(1000), 339.2692(927), 321.2585(648), 215.1795(277), 211.1481(175), 201.1638(406),  185.1328(160), 177.1271(183), 175.1481(296), 161.1324(389), 159.1167(201), 158.0812(277),  149.1326(191), 147.1169(255), 135.1168(254), 133.1012(202), 76.0413(224) | 432.3111 | C_26_H_41_O_4_N | 1030759 | 88483 |
| 69.09^a,b^ | MS2[448.3@35]: 430.2958(239), 373.2737(51), 355.2640(1000), 337.2533(242), 319.2425(80), 229.1592(58), 175.1111(77), 76.0407(129) | 448.3061 | C_26_H_41_O_5_N | 343166 | 38918 |
| 70.91 | MS2[1042.5@-92]: 437.1650(664), 365.1462(224), 324.1352(336), 323.1303(1000), 213.0952(224), 201.0928(440), 187.0776(224), 135.0467(664), 117.0204(224), 113.0259(888), 109.0306(224) | 1042.4894 | C_50_H_77_O_22_N | 15138 | 1843 |
| 75.56^a^ | MS2[480.3@-92]: 426.2374(506), 408.2285(338), 206.0470(169), 124.0062(831), 121.0305(662), 106.9824(1000) | 480.2785 | C_26_H_43_O_5_NS | 92679 | 5449 |

^a^ The retention time and detailed HRMS^2^ data of ZSIN.

^b^ The retention time in positive base peak of high resolution mass spectrum.
